# Bimetallic NiFe Nanoparticles Supported on CeO_2_ as Catalysts for Methane Steam Reforming

**DOI:** 10.1021/acsanm.3c00104

**Published:** 2023-05-02

**Authors:** Andrea Braga, Marina Armengol-Profitós, Laia Pascua-Solé, Xavier Vendrell, Lluís Soler, Isabel Serrano, Ignacio J. Villar-Garcia, Virginia Pérez-Dieste, Núria J. Divins, Jordi Llorca

**Affiliations:** † Institute of Energy Technologies, Universitat Politècnica de Catalunya, EEBE, Eduard Maristany 10-14, 08019 Barcelona, Spain; ‡ Department of Chemical Engineering, Universitat Politècnica de Catalunya, EEBE, Eduard Maristany 10-14, 08019 Barcelona, Spain; § Barcelona Research Center in Multiscale Science and Engineering, Universitat Politècnica de Catalunya, EEBE, Eduard Maristany 10-14, 08019 Barcelona, Spain; ∥ ALBA Synchrotron Light Source, Carrer de la Llum 2-26, 08290 Cerdanyola del Vallès Barcelona, Spain

**Keywords:** ambient-pressure X-ray photoelectron spectroscopy, bimetallic catalyst, cerium oxide, hydrogen, mechanochemistry, methane steam reforming, nickel-iron

## Abstract

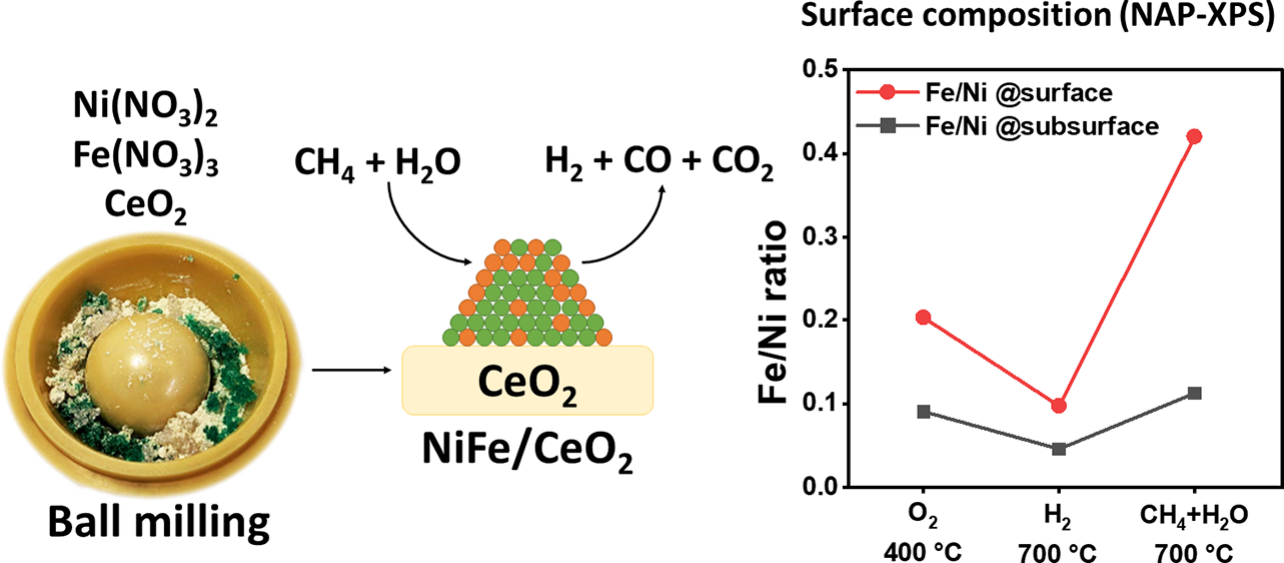

Ni-Fe nanocatalysts supported on CeO_2_ have been prepared for the catalysis of methane steam reforming (MSR) aiming for coke-resistant noble metal-free catalysts. The catalysts have been synthesized by traditional incipient wetness impregnation as well as dry ball milling, a green and more sustainable preparation method. The impact of the synthesis method on the catalytic performance and the catalysts’ nanostructure has been investigated. The influence of Fe addition has been addressed as well. The reducibility and the electronic and crystalline structure of Ni and Ni-Fe mono- and bimetallic catalysts have been characterized by temperature programmed reduction (H_2_-TPR), in situ synchrotron X-ray diffraction (SXRD), X-ray photoelectron spectroscopy (XPS), and Raman spectroscopy. Their catalytic activity was tested between 700 and 950 °C at 108 L g_cat_^–1^ h^–1^ and with the reactant flow varying between 54 and 415 L g_cat_^–1^ h^–1^ at 700 °C. Hydrogen production rates of 67 mol g_met_^–1^ h^–1^ have been achieved. The performance of the ball-milled Fe_0.1_Ni_0.9_/CeO_2_ catalyst was similar to that of Ni/CeO_2_ at high temperatures, but Raman spectroscopy revealed a higher amount of highly defective carbon on the surface of Ni-Fe nanocatalysts. The reorganization of the surface under MSR of the ball-milled NiFe/CeO_2_ has been monitored by in situ near-ambient pressure XPS experiments, where a strong reorganization of the Ni-Fe nanoparticles with segregation of Fe toward the surface has been observed. Despite the catalytic activity being lower in the low-temperature regime, Fe addition for the milled nanocatalyst increased the coke resistance and could be an efficient alternative to industrial Ni/Al_2_O_3_ catalysts.

## Introduction

Nowadays, hydrogen is mainly produced from steam reforming of methane (MSR, reaction 1),^1^ in which methane reacts with water to generate a mixture of H_2_ and CO. This mixture is called synthesis gas (syngas),^2^ as it is the starting reactant mixture to synthesize chemical products such as methanol or for the Fischer–Tropsch process. The process is highly endothermic, requiring high temperatures for operation. Together with MSR, the water-gas-shift reaction (WGSR, reaction 2) takes place, in which CO further reacts with H_2_O to form CO_2_ and more H_2_.
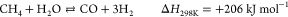
1
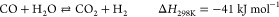
2

Active and robust catalysts are needed to activate methane. Industrially, Ni/Al_2_O_3_ is the most used catalyst^3^ because Ni possesses similar catalytic properties to noble metals at a cheaper price and it is more abundant,^4^ but it has been identified as a strategic raw material by the European Commission.^5^ Nevertheless, Ni catalysts suffer from high rates of carbon formation, passivation and oxidation, formation of inactive NiAl_2_O_4_, sintering, and sulfur poisoning.^3,6^ Several strategies have been devised to increase the catalytic performance and the stability of Ni-based catalysts, such as modifying the Al_2_O_3_ support,^7^ adding alkali metals as promoters,^8^ doping with rare-earth elements,^9,10^ or forming mixed oxides with MgO, ZnO, and La_2_O_3_.^11,12^ Besides Al_2_O_3_, other oxide materials have been used as supports, such as MgAl_2_O_4_,^13,14^ SiO_2_,^15^ MgO,^16^ ZrO_2_,^17^ CeO_2_,^18^ perovskites,^19,20^ or mixtures of these oxides,^21^ showing that a strong interaction between Ni and the oxide material is important to prevent sintering and that a high surface basicity is important for CO and CO_2_ activation to reduce coke deposition.^3,11^

Another strategy to enhance the catalytic activity and stability of Ni-based catalysts is to form bimetallic alloys.^22,23^ Noble metals such as Pt, Pd, Ir, Ru, and Rh have been used to enhance the physicochemical properties of Ni.^14,24−27^ Early works showed that small amounts of Rh (0.05–0.2%) improved the activity of a Ni/Al_2_O_3_ catalyst leading to higher Ni dispersion and reducibility.^24^ Noble metals are very effective at activating H_2_ molecules and increasing the reducibility of NiO via hydrogen spillover, which can lead to the self-reduction, i.e., self-activation of Ni-based catalysts.^28−30^ This property is also important to keep Ni reduced during the reforming reaction. Gold and Ag promotion increased catalysts’ stability and coke resistance but resulted in a lower catalytic activity^9,31,32^ because these elements interact strongly with low-coordinated Ni atoms on stepped surfaces, which are the most active sites for both CH_4_ activation and carbon formation.^9^ Among non-noble metals, Ni-based bimetallic catalysts with Cu,^33,34^ Co,^17^ and Mo^35^ have been studied. Copper showed positive effects on the stability of Ni catalysts due to the blockage of the most active sites for coke formation.^34^ Cobalt addition to Ni led to stable catalysts thanks to the total suppression of carbon formation in the presence of oxygenated CoO_*x*_ species.^17^ The addition of Mo to Ni/Al_2_O_3_ decreased the reducibility and dispersion of Ni, but surprisingly, the catalytic activity was still high because of the electronic donation of Mo to Ni resulting in increased electronic density and more favorable conditions for MSR.^35^

The synergy between Ni and Fe supported on nonreducible supports has also been investigated.^33,36−38^ Iron is weakly active for the MSR reaction,^4^ whereas it is very active for the WGSR and it easily forms alloys with Ni. Early works on NiFe bimetallic catalysts for MSR showed increased catalyst stability due to the oxophilicity and redox properties of Fe that facilitated carbon oxidation.^36^ In addition, increased H_2_ production at lower temperatures was observed due to the equilibrium shift of the WGSR.^36^ NiFe catalysts have been prepared by exsolution of Fe from mixed oxides such as Fe-containing perovskites and vermiculites.^37,38^ Besides MSR, NiFe bimetallic catalysts have been extensively used for other reforming reactions and methanation reactions.^39−41^ Generally, three main effects are observed upon Fe addition: (i) smaller NiO nanoparticles after calcination and higher Ni dispersion after reduction being achieved, (ii) formation of mixed spinel species such as (Ni,Fe)Al_2_O_4_ that are more reducible than NiAl_2_O_4_, and (iii) gasification of carbonaceous species due to Fe redox properties. Fe/(Ni + Fe) weight ratios of 0.1–1 showed the best equilibrium between increased stability, increased dispersion, oxidation of Ni active phase, and decreased activity due to Ni substitution with the less active Fe.^39^ The effect of Fe as a promoter in Ni-Mg-Al hydrotalcites and Ni/ZrO_2_^42,43^ was studied, and the conclusion was that Fe has similar redox properties to CeO_2_ (ceria), which is a well-known redox oxide material with high oxygen mobility and high water activation ability.^23,44^ The use of ceria as a support material has increased considerably in the last decades owing to the synergy obtained between ceria and the supported metals thanks to the development of strong metal–support interactions,^45^ which affect the reorganization of metallic nanoparticles on its surface. Nevertheless, to the best of our knowledge, NiFe/CeO_2_ catalysts have not been studied for the MSR reaction. Thus, in this work, we investigate the synergy between Ni and Fe nanoparticles when they are supported on ceria and their interaction with the support. Additionally, we synthesized our nanocatalysts by mechanochemistry, which is a synthesis method underexploited for the preparation of solid catalysts and that is receiving increasing attention. Mechanochemistry is a simple and eco-friendly method because no solvents are used, thus avoiding the production of large amounts of solvent waste and of subsequent treatments at high temperatures. The mechanochemical synthesis of catalysts can be readily scaled up to meet industrial needs. Additionally, during the milling process, unconventional architectures can be obtained, such as high defect concentrations, modified interaction between the components, or new materials’ arrangements.^46,47^

**Scheme 1:**
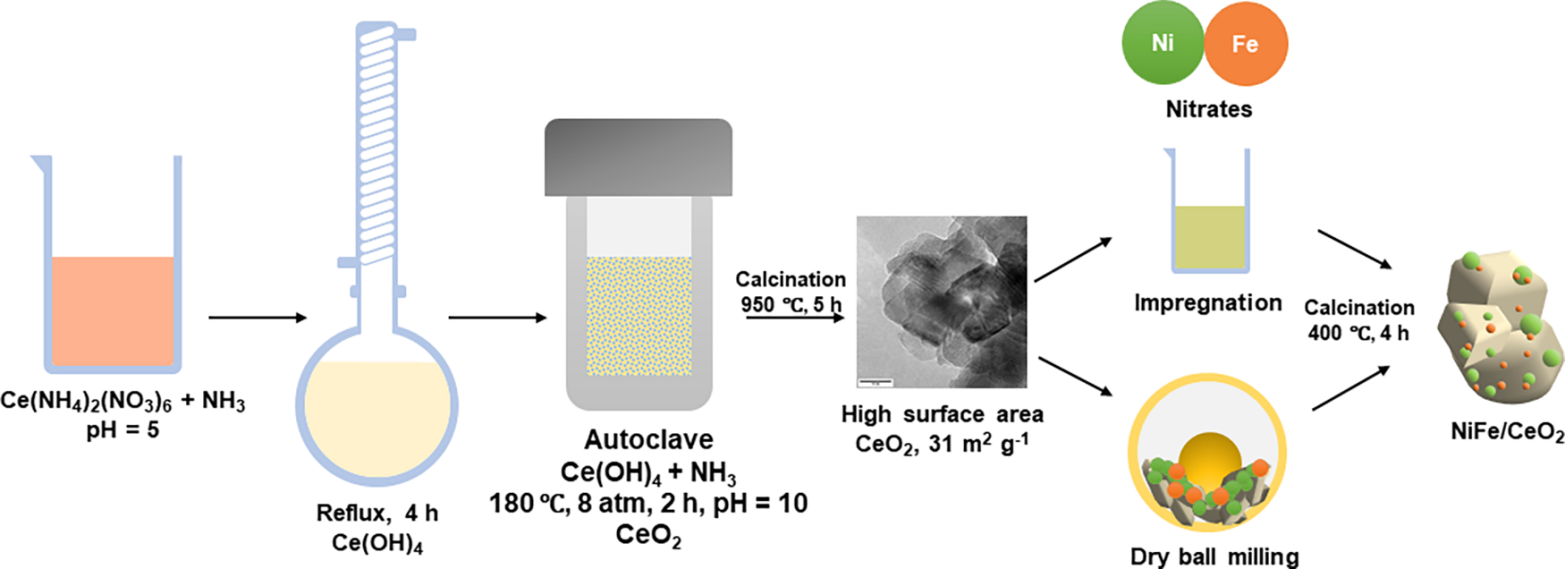
Schematic Illustration of the Synthesis Procedures Followed to Prepare the Investigated NiFe/CeO_2_ Nanocatalysts by Incipient Wetness Impregnation and Ball Milling

In this work, we investigate a series of mono- and bimetallic NiFe nanocatalysts supported on ceria prepared by conventional impregnation methods and mechanochemistry^47−49^ to explore the combination of Ni, Fe, and CeO_2_ for the catalysis of methane steam reforming reaction (Scheme 1). The possibility to obtain nanocatalysts synthesized via a greener and easier synthesis method, namely, mechanochemistry,^46^ compared to traditional impregnation is also addressed.

## Experimental Section

### Catalyst Preparation

Cerium dioxide (CeO_2_) with a high surface area was prepared by a thermal hydrolysis process followed by a hydrothermal treatment.^50,51^ Ce(NH_4_)_2_(NO_3_)_6_ (1 mol L^–1^) was prepared in deionized water under vigorous stirring for 30 min. A volume of 125 mL of NH_3_ (0.35 mol L^–1^) was added dropwise to 65 mL of the cerium solution while stirring. The solution was kept under reflux for 4 h; as soon as the boiling point was reached, a yellow precipitate of Ce(OH)_4_ seeds formed. The precipitate was separated from the solution by centrifugation and transferred to 50 mL of 2.0 mol L^–1^ NH_3_ in a Teflon vessel. The hydrothermal treatment was carried out at 180 °C for 2 h in a stainless-steel autoclave. The final CeO_2_ product was filtered and washed with deionized water, dried at 110 °C for 24 h, and calcined at 950 °C for 5 h in air. Reference cerium oxide was prepared with the common ammonia precipitation method as follows: a solution of NH_3_ 28 wt % was added dropwise to a 10 wt % Ce(NO_3_)_3_ water solution reaching a pH of 9.5 while stirring, and a purple suspension was obtained and aged under stirring overnight. Overnight, the solution oxidized, turning yellow. After filtrating and washing with water, the powder was dried at 110 °C for 24 h and calcined at 950 °C for 5 h in air.

Catalysts were synthesized via two methods, namely, incipient wetness impregnation (IWI) and mechanochemically (ball milling, BM). The total metal loading was fixed to 9 wt %. Samples are named Ni/CeO_2_, Fe_0.1_Ni_0.9_/CeO_2_, and Fe_0.2_Ni_0.8_/CeO_2_, where the subscripts refer to the atomic percentage of Ni and Fe in the metallic phase and -IWI or -BM suffixes refer to the two synthesis methods. For the IWI samples, given amounts of Fe(NO_3_)_3_·9H_2_O and Ni(NO_3_)_2_·6H_2_O were dissolved in a 1:1 v/v water/ethanol mixture. Nickel and iron nitrates were impregnated simultaneously on cerium oxide in multiple steps followed by drying steps in air at 110 °C for 1 h. The final product was dried at 110 °C for 24 h and calcined at 400 °C for 4 h. BM samples were prepared in a FRITSCH Mini-Mill Pulverisette 23, with a 15 mL ZrO_2_ jar and a single 10 g ball (Ø = 15 mm), by directly placing CeO_2_ and the corresponding amount of Ni and/or Fe nitrate precursors in the jar. The milling parameters were time (*t*) = 15 min of milling duration, oscillation frequency (*f*) = 30 Hz, and ball-to-powder ratio (BPR) = 20. The product was dried at 110 °C for 1 h and calcined in air at 400 °C for 4 h.

### Catalyst Characterization

The specific surface area of CeO_2_ was measured by BET in an ASAP-2020 instrument (Micromeritics). Nitrogen was used as the adsorbent molecule at −196 °C, and the sample was outgassed at 200 °C for 2 h before the measurement. The effective metal loading of Ni and Fe was measured by inductively coupled plasma optical emission spectroscopy (ICP-OES) using a Perkin Elmer Optima 2300 spectrometer. For the ICP-OES measurements, 50 mg of the sample was digested in a mixture of 2 mL of HNO_3_, 2 mL of H_2_SO_4_, and 500 mg of NH_4_Cl, with the digestion assisted with microwave heating at 240 °C for 30 min.

The reduction properties of NiFe catalysts were measured by H_2_ temperature-programmed reduction (H_2_-TPR) using a Chemstar-TPX instrument equipped with a thermal conductivity detector (TCD). About 50 mg of the sample was initially pretreated in Ar (30 mL min^–1^) heating from room temperature to 350 °C (15 °C min^–1^), holding for 30 min, and cooling to 100 °C, and then the TPR was performed with 10% H_2_/Ar (50 mL min^–1^) from 100 to 850 °C (10 °C min^–1^).

The morphology of the as-prepared catalysts was studied with transmission electron microscopy (HRTEM) with an FEI TECNAI F20 microscope at 200 kV. Samples were dispersed in methanol and drop casted on carbon-covered copper grids.

Raman spectroscopy was performed using a Renishaw IN-VIA apparatus with a 532 nm laser at 1 mW power and a 20× objective to assess the composition of deposited carbon species on spent catalysts. Carbon D and G bands were deconvoluted with a set of five peaks in CasaXPS 2.3.25 using the lineshape GL(90).^52^ Spent catalysts were separated from the SiC used for catalytic testing by sieving the catalytic bed. Several spectra in different regions were collected for each sample to properly describe the properties of the spent catalyst.

Thermogravimetric analyses (TGAs) were performed using a Q500 instrument from TA Instruments. The analyses were performed from room temperature to 900 °C (10 °C min^–1^ ramp) with 50 mL min^–1^ of synthetic air.

The electronic properties of the catalysts’ surface were measured by X-ray photoemission spectroscopy (XPS) using a SPECS instrument equipped with a Mg X-ray source at 300 W and a Phoibos 150 MCD-9 detector with a pass energy of 20 eV. High-resolution spectra were acquired in steps of 0.1 eV. XPS measurements were performed on calcined catalysts (as-prepared catalysts) as well as after the reaction tests performed at 950 °C in the laboratory. The instrument is also equipped with an in situ high-pressure cell (HPC), where chemical reactions can be performed up to 20 bar and the samples can be transferred to the analysis chamber under ultra-high vacuum (UHV). In situ reductions were performed on calcined catalysts in the HPC with 20 mL min^–1^ of 10% H_2_/Ar at 350 °C for 30 min, and the corresponding spectra were acquired after the treatments. Surveys and high-resolution Ce 3d + Ni 2p, Ni LMM (spectra not shown), Fe 2p, O 1s, and Ce 4s + C1s spectral regions were measured. The energy calibration to compensate for the charge was done considering the binding energy (BE) of the Ce 3d_3/2_ u”’ peak at 916.9 eV.^53^ Data analysis was performed with CasaXPS 2.3.25 using an asymmetric lineshape LF(0.95,1.25,20,20) for the metallic Ni main peak and GL(50) lineshapes for nickel oxides, which were fitted following a previously reported model for NiO and Ni(OH)_2._^54^ A set of three peaks for metallic Ni, five peaks for NiO and its satellite, and three peaks for Ni(OH)_2_ and its satellite for the Ni 2p_3/2_ region were used following the above-mentioned model. The Ce 3d region was deconvoluted following the model described in refs (53, 55) with a set of four peaks for Ce^3+^ and six peaks for Ce^4+^ to account for the spin–orbit splitting multiplets. A spline Shirley background for the Ce 3d and the Ni 2p_1/2_ region and a Shirley background for the Ni 2p_3/2_ region were used to account for the complex overlapping regions of Ni 2p and Ce 3d. In the overlapping portion between Ce 3d_5/2_ and Ni 2p_1/2_ (between 878 and 890 eV), peaks corresponding to Ni 2p_1/2_ were considered to obtain a complete fitting of the measured data, but the relative concentration of Ni species was calculated using only the Ni 2p_3/2_ region.

Near-ambient pressure XPS (NAP-XPS) was performed on sample Fe_0.1_Ni_0.9_/CeO_2_-BM at the NAPP end station of beamline BL-24 CIRCE of the ALBA synchrotron, which is equipped with a Phoibos NAP150 electron analyzer from SPECS. The sample was pelletized and mounted on a sample holder equipped with a thermocouple in direct contact with its surface. The sample was heated up by using an infrared laser. High-resolution spectra were acquired with 0.1 eV energy step and 10 eV pass energy. The gases were dosed in the analysis chamber using independent mass flow controllers. A dynamic pressure of 1 mbar was kept during the whole in situ experiment controlled with an automatic valve. The composition of the gaseous atmosphere inside the analysis chamber was monitored with a mass spectrometer connected to the second pumping stage of the detector. The series of experiments consisted of (i) dosing 10 mL min^–1^ of O_2_ from room temperature to 400 °C (10 °C min^–1^) and holding for 30 min at 400 °C to clean the sample surface of adventitious carbon. Then, the sample was cooled to 100 °C, and O_2_ was removed. (ii) A reduction was performed with 10 mL min^–1^ of H_2_ from 100 to 700 °C (10 °C min^–1^), holding for 30 min. (iii) The methane steam reforming mixture was dosed by flowing 1.25 mL min^–1^ of CH_4_ and 2.5 mL min^–1^ of H_2_O (steam-to-carbon ratio (S/C) = 2) in the analysis chamber at 700 °C. At each of these three steps, a low-resolution survey and high-resolution Ce 3d + Ni 2p, Fe 2p, C 1s, and O 1s spectral regions were measured. Two X-ray photon energies were chosen for each region to generate photoelectrons with two kinetic energies (450 and 215 eV) for all regions, thus allowing probing the sample’s surface at two different depths. The inelastic mean free path (IMFP) at 215 eV for Ni 2p and Fe 2p is approximately 0.6 nm, whereas at 450 eV, it is about 1 nm (see Table S1).^56^ Additionally, to perform the energy correction, all regions were acquired with 1335 eV photon energy. The energy correction was done again considering the theoretical BE of the Ce 3d_3/2_ u”’ peak to 916.9 eV. The shift observed for this peak was used to correct for energy the other regions acquired with the same photon energy (1335 eV), and this information was also used to calibrate the spectra acquired with the other photon energies. The data analysis was performed with CasaXPS 2.3.25. The same peak models as for the UHV-XPS measurements were used for the NAP-XPS experiments, except for the main line of metallic Ni, where a lineshape LF(0.8,1.57,80,50) was used. The atomic fraction quantification of Ni, Fe, Ce, and O was done by correcting the measured areas with the relative sensitivity factors (RSFs), where the corresponding ionization cross section for each element and energy was calculated using the data reported in ref (57), and the photon flux at each photon energy and the analyzer transmission function were considered.

Structural properties of NiFe catalysts were investigated by in situ synchrotron X-ray diffraction (SXRD) at the BL-04 MSPD beamline at ALBA synchrotron using a wavelength of 0.413 Å (30 keV of photon energy). About 5 mg of the powder sample was loaded inside quartz capillaries and mounted in a sample holder connected to a gas dosage system. Diffractograms were recorded during the temperature-programmed reductions (TPRs) under a 5% H_2_/Ar (5 mL min^–1^) flow from room temperature to 700 °C (10 °C min^–1^) and holding for 30 min at 700 °C. After the reduction, the MSR mixture with a steam-to-carbon ratio of about 2 was achieved by flowing 5 mL min^–1^ of 1% CH_4_/N_2_ through a water saturator at room temperature. Diffractograms of samples under MSR conditions were measured at 700, 800, and 950 °C and during the heating ramps (10 °C min^–1^). Whole pattern Rietveld refinements were performed on XRD diffractograms with GSAS-II and OriginPro 9.0 for peak fitting using pseudo-Voigt-I functions.

### Catalytic Testing

Methane steam reforming tests were performed by mixing the catalysts with SiC in quartz reactors (fixed bed reactor, inner diameter 8 mm) at atmospheric pressure. Catalysts were activated in 10% H_2_/N_2_ (50 mL min^–1^) from room temperature to 700 °C (10 °C min^–1^), keeping this temperature for 30 min. After the reduction, the reaction mixture that was composed of CH_4_/H_2_O/N_2_ in the ratio 1:2:1.5 (S/C = 2) was dosed. Water was injected with an HPLC pump (Knauer) and vaporized at 120 °C. The influence of temperature on the catalytic activity was investigated using 125 mg of samples diluted with SiC to a total bed volume of 1.25 cm^3^ at temperatures ranging from 700 to 950 °C (50 °C steps, 45 min at each step). This temperature range was selected to obtain high methane conversions. Additionally, below that temperature range, there can be higher selectivity for carbon deposition. For these experiments, a flow-to-weight ratio (F/W) = 108,000 mL g_cat_^–1^ h^–1^ and space velocity (GHSV) = 10,800 h^–1^ were chosen because they are common spatial velocities reported in the literature. For a mass of 125 mg of catalyst, a total inlet flow of 225 mL min^–1^ was required to obtain the mentioned F/W and GHSV.

The effect of varying the flow-to-weight ratio was studied with 50 mg of the sample diluted with SiC to a total bed volume of 0.5 cm^3^ in the F/W range between 54,000 and 415,800 mL g_cat_^–1^ h^–1^ (GHSV = 5400–41,580 h^–1^) at a fixed temperature of 700 °C, staying for 45 min at each step and reaching stable catalytic activity. The reaction products were analyzed using a micro-GC (Agilent Technologies 3000A Micro GC) equipped with a molecular sieve 5A and a PoraPlotU column. The carbon balance of all the experiments was calculated, and values within 100 ± 3% were obtained. The deviations observed from 100% were due to the experimental error of the experimental setup (gas microchromatograph calibration, mass flow controllers’ calibration, accuracy of the total outlet flow calculated).

Methane conversion (*X*_CH4_, eq 3, where *ṅ* are the molar flowrates), the product’s selectivity (*S_i_*, with *i* = H_2_, CO, CO_2_, eq 4), and the H_2_ yield (*Y*_H2_, eq 5) were defined as follows:
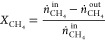
3
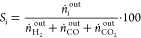
4
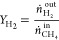
5

The hydrogen production rate (*r*_H_2__, eq 6) was calculated as follows:
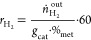
6where *ṅ*_H_2__^out^ is the H_2_ molar flow at the outlet in mol min^–1^ and the metal percentage indicates the total amount of metal, i.e., Ni + Fe.

## Results

### As-Prepared Catalysts’ Characterization

After calcining the hydrothermally prepared CeO_2_ at 950 °C for 5 h, the surface area was 31.3 ± 0.3 m^2^ g^–1^, which is lower compared to the more common Al_2_O_3_ or MgAl_2_O_4_ supports. Nevertheless, this surface area is larger than the surface area obtained for CeO_2_ prepared via common ammonia precipitation, which showed a surface area of about 0.4 ± 0.1 m^2^ g^–1^ after calcination under the same conditions, which is about 78 times lower compared with the high surface area ceria. This shows that the hydrothermal synthesis resulted in a larger surface area, as expected. The composition of the catalysts was measured by ICP-OES; the metal loading ranged between 7.8 and 8.9 wt % (Table S2). The Fe/Ni mass ratios were very close to the nominal ones, being 0.11 for Fe_0.1_Ni_0.9_/CeO_2_ samples and 0.25 for Fe_0.2_Ni_0.8_/CeO_2_ samples.

**Figure 1:**
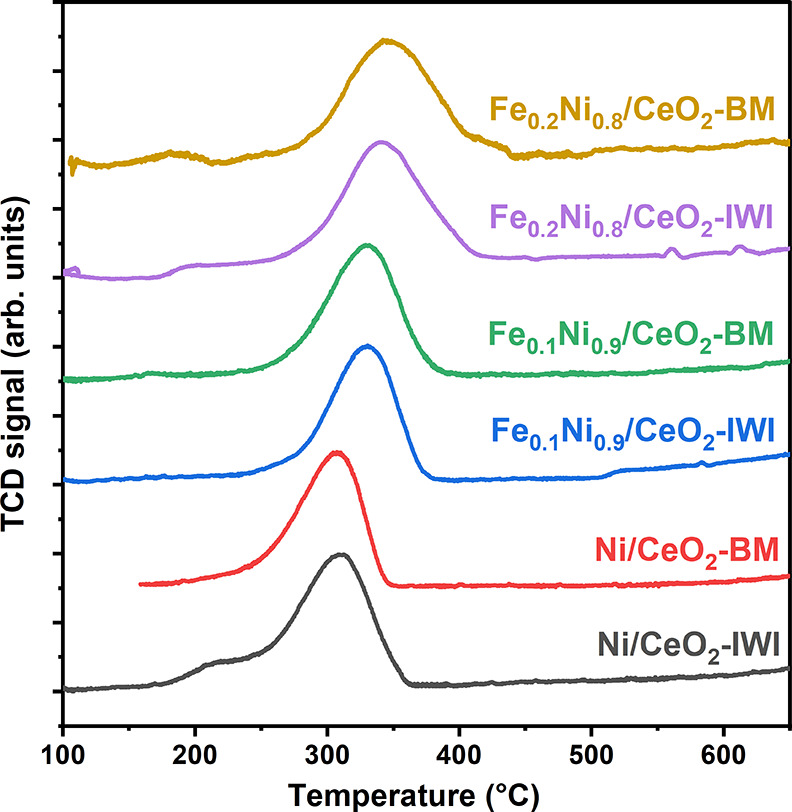
H_2_-TPR profile of NiFe/CeO_2_ catalysts.

The TPR profiles displayed in Figure 1 show the reducibility of the NiFe/CeO_2_ systems. The presence of single peaks could be ascribed to the direct reduction of both NiO and iron oxides to form the metallic Ni or NiFe alloy^58^ (see also below the in situ XRD discussion). The reduction of monometallic catalysts occurred at about 305 °C, whereas the substitution of 10 at % of Ni with Fe in the active phase increased the temperature to about 330 °C, and the further substitution of Ni with 20 at % of Fe further increased it to about 340 °C. This is due to the higher temperature required to reduce iron oxides compared to nickel oxides and to the interaction between Ni and Fe.^58^ Broader asymmetric peaks for Fe_0.2_Ni_0.8_/CeO_2_ catalysts can be associated with the initial reduction of Fe_2_O_3_ species to Fe_3_O_4_ followed by the reduction of NiO and FeO_*x*_ together.^59,60^

**Figure 2:**
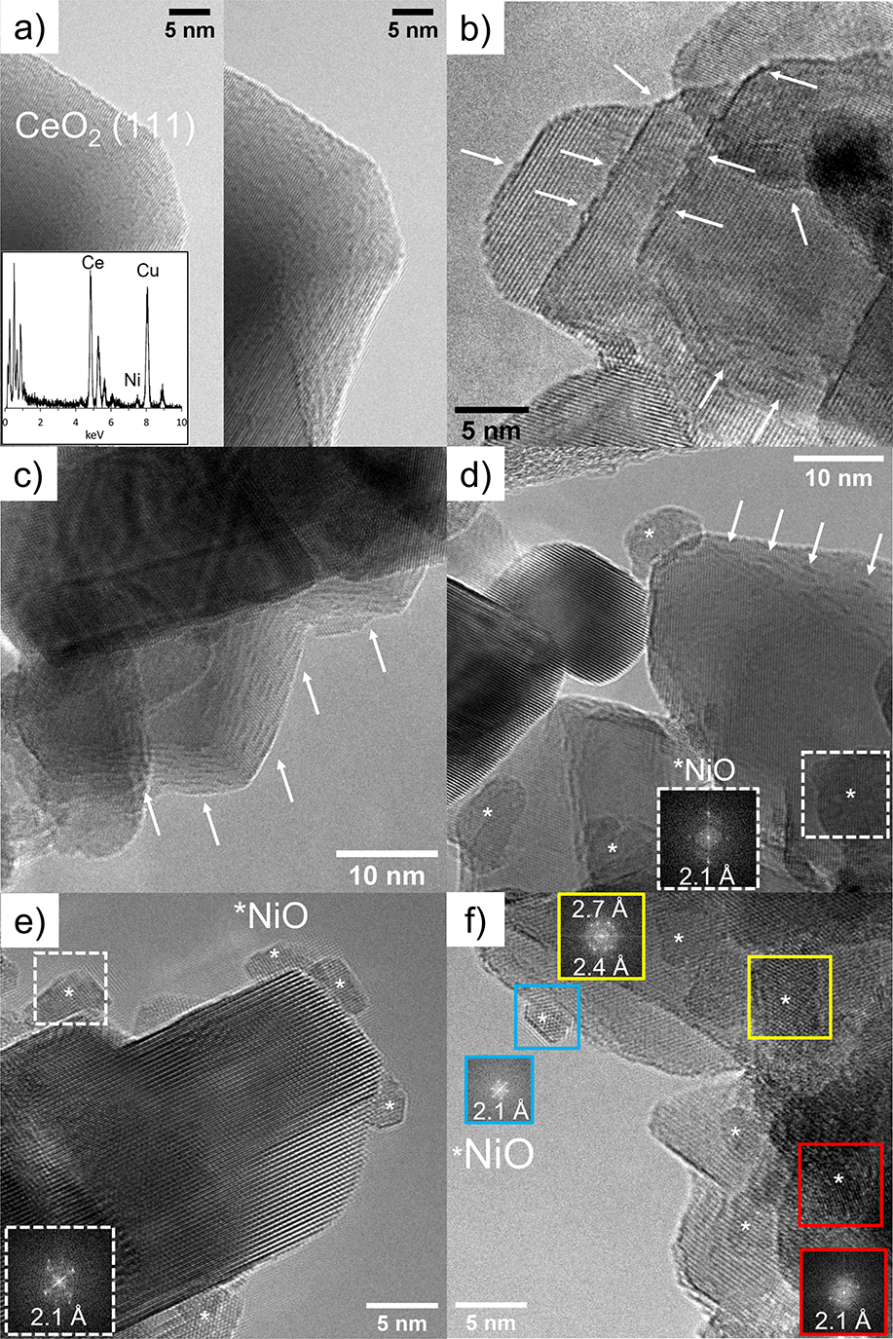
HRTEM of (a) Ni/CeO_2_-IWI, (b) Ni/CeO_2_-BM, (c) Fe_0.1_Ni_0.9_/CeO_2_-IWI, (d) Fe_0.1_Ni_0.9_/CeO_2_-BM, (e) Fe_0.2_Ni_0.8_/CeO_2_-IWI, and (f) Fe_0.2_Ni_0.8_/CeO_2_-BM. The NiO nanoparticles are marked with asterisks. The Fourier transform spots at 2.1 and 2.4 Å correspond to NiO (111) and (200) fringes, whereas the reflection at 2.7 Å in panel f corresponds to the CeO_2_ (200) crystallographic plane. The Cu signal seen in the EDX spectrum displayed in the inset of panel a originates from the TEM Cu grid.

The morphology and phases of the Ni/CeO_2_ and FeNi/CeO_2_ catalysts were studied by HRTEM, and representative images are shown in Figure 2 and Figure S1. Ceria nanocrystals appeared in highly crystalline formations, showing mainly lattice fringes corresponding to CeO_2_ (111), (200), and (220) planes at 3.1, 2.7, and 1.9 Å, respectively. No differences in the ceria crystallites were observed between IWI and BM samples. For both monometallic samples, the surface of ceria crystallites was covered with sub-nanometric clusters (Figure 2a,b and Figure S1a,b). Energy-dispersive X-ray spectroscopy (EDX) measurements performed on the region displayed in Figure 2a confirmed the presence of Ni on ceria when these clusters were detected, as shown in the inset of Figure 2a. In both bimetallic Fe_0.1_Ni_0.9_/CeO_2_ catalysts, sub-nanometric row-like structures were identified on the ceria crystallites, marked with arrows in Figure 2c,d. EDX measurements performed on the regions displaying the row-like structures revealed the presence of Ni (Figure S1c), whereas Ni signals were not detected in regions where only CeO_2_ fringes were visible. These results show that Ni was well-dispersed, forming sub-nanometric structures on the ceria surface. Together with these small clusters, the presence of NiO NPs with sizes ranging between 10 and 20 nm was observed (Figure 2d, Figure S1a,b,d,e) for both Ni/CeO_2_ and Fe_0.1_Ni_0.9_/CeO_2_ catalysts, indicating the presence of particles with a wide size distribution. In Fe_0.2_Ni_0.8_/CeO_2_ samples, more defined NiO NPs were observed, and the smaller clusters were not identified. Because of the low amount of Fe, it was not possible to observe or detect any iron-oxide-related fringes in the bimetallic catalysts. The size of the NiO nanoparticles ranged between 5 and 20 nm.

### SXRD Measurements Ex Situ of As-Prepared Catalysts and In Situ Reduction and Methane Steam Reforming

**Figure 3:**
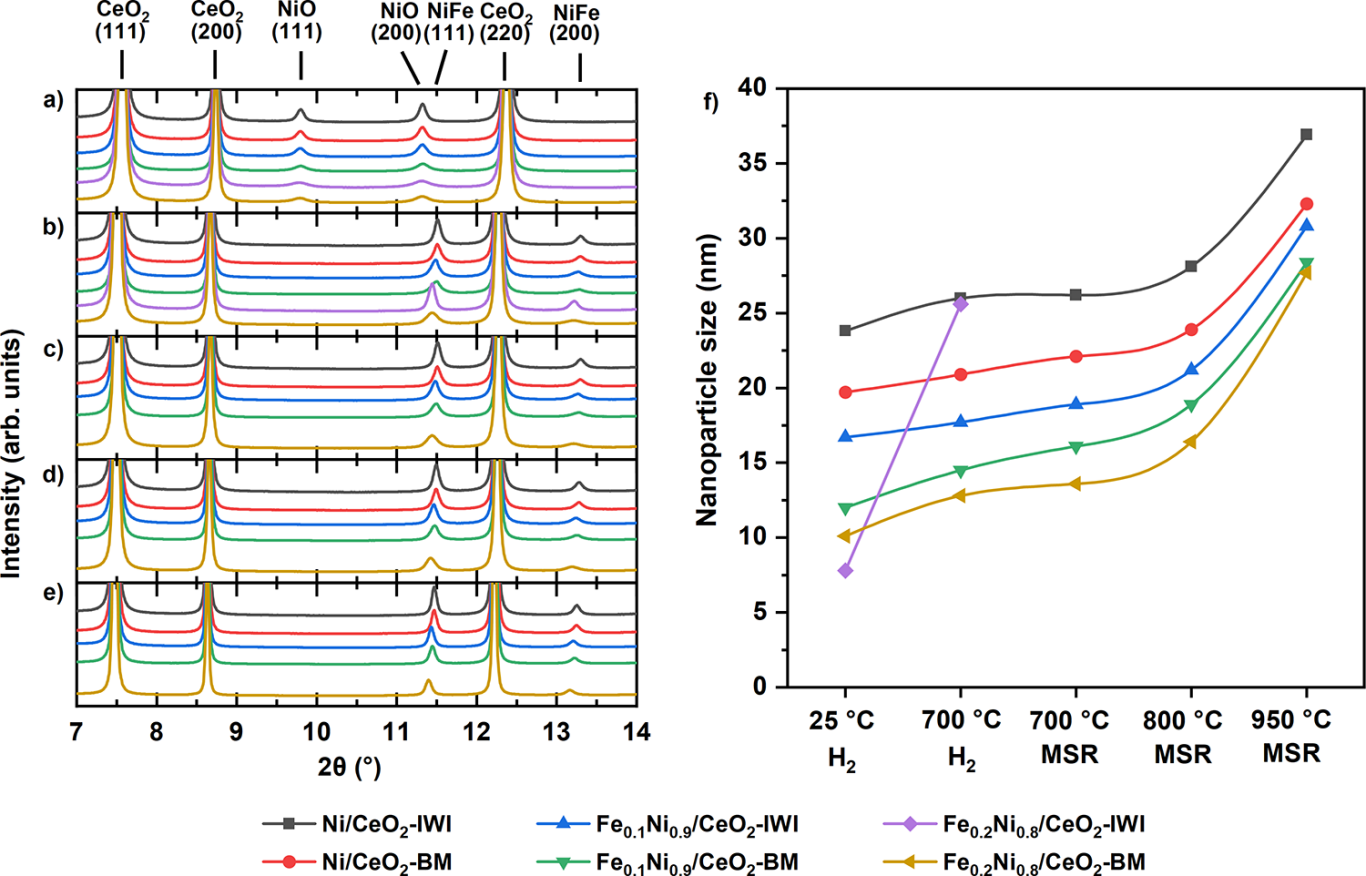
In situ SXRD diffractograms of Ni/CeO_2_ and FeNi/CeO_2_ samples (a) as-prepared at room temperature, (b) under H_2_ at 700 °C for 30 min, (c) after 60 min at 700 °C under MSR, and (d, e) at 800 and 950 °C under MSR. (f) NiO, Ni, and NiFe size at different stages as obtained from Rietveld refinement. λ = 0.413 Å.

In Figure 3a, the diffractograms of as-prepared samples, after 30 min of reduction at 700 °C (Figure 3b) and under MSR at 700 (after 60 min, Figure 3c), 800, and 950 °C (after 30 min, Figure 3d,e), are reported. Figure 3f displays the size of NiO, Ni, and NiFe estimated by Rietveld refinement analysis (see Figure S2). All as-prepared samples showed reflections of CeO_2_ and NiO, whereas no Fe oxide reflections were observed (Figure 3a). The NiO crystallite size was 23.8 and 19.7 nm, respectively, for the monometallic Ni/CeO_2_-IWI and Ni/CeO_2_-BM samples, which decreased upon Fe addition to 7.8 and 10.1 nm for samples containing 20% Fe in the active phase (Table S3).

The synthesis method also influenced the NiO crystallite size, and it was smaller for the BM catalysts (Figure 3f). After the reduction step at 700 °C for 30 min, all samples showed complete reduction to Ni or NiFe alloy. As seen in Figure 3b, only one peak centered around 11.5° was visible after reduction, which is attributed to the formation of Ni and NiFe alloys. The inclusion of Fe in the Ni lattice is confirmed by the shift of the peak toward lower 2θ values as the amount of Fe increases. The lattice parameters of NiO, Ni, and NiFe obtained from the Rietveld refinements are reported in Table S4 and Figure S3. Figure S4 and Table S5 report the results for CeO_2_. The results from the Rietveld refinements also confirm the inclusion of Fe in the Ni lattice, as the cell parameters increase accordingly to the amount of Fe added, demonstrating the inclusion of Fe atoms with larger atomic radius inside the Ni lattice.^61^

After switching to MSR conditions, the bulk of the active phase for all the samples remained metallic (Figure 3c–e). After 1 h at 700 °C under MSR, the decreasing crystallite size trend with the addition of Fe was maintained, and no sintering was detected. The lattice parameters of Ni and NiFe were unvaried compared to the values after reduction, confirming the stability of the alloys. The size of Ni and NiFe nanoparticles after 30 min at 800 °C under MSR reaction mixture increased by 2.8 nm on average, with the increase being larger for the bimetallic samples. The increasing trend of the lattice parameter with Fe addition is preserved under MSR at 800 and 950 °C (Figure S3). During MSR at 950 °C, the Ni and NiFe crystallite size sintering accelerated with an average size increase of 9.5 nm. Comparing the Ni and NiFe crystallite size before and after the exposure to MSR, a size increase of 11 nm for the monometallic samples and 14 nm for the bimetallic samples was observed, with the largest sintering occurring between 800 and 950 °C (Figure 3f, Table S3).

### XPS of As-Prepared Catalysts and Reduced In Situ inside the HPC

**Figure 4:**
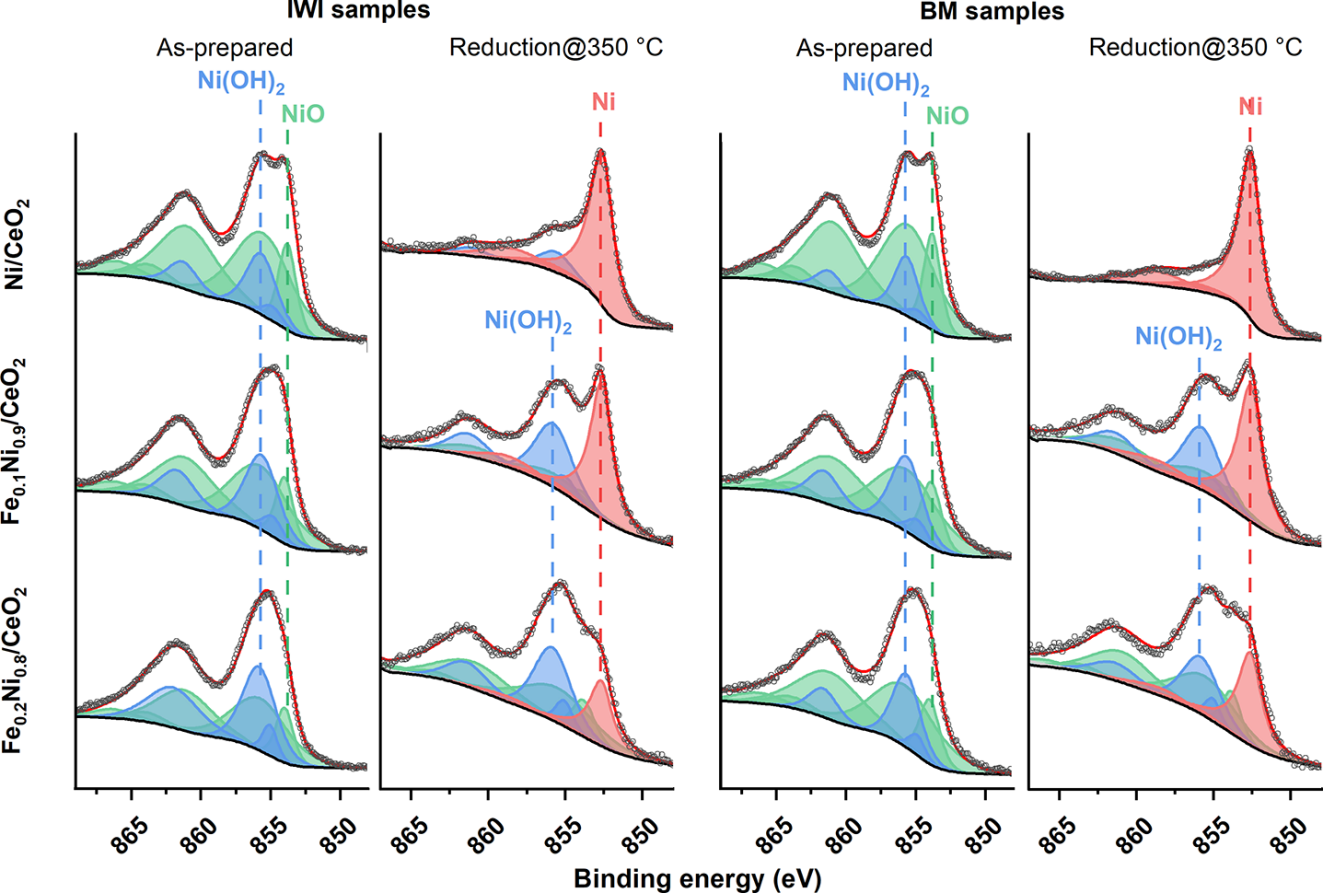
Ni 2p_3/2_ XPS spectra of the as-prepared catalysts and after in situ reduction inside the high-pressure cell at 350 °C for 30 min with 20 mL min^–1^ of 10% H_2_/Ar. The spectra are normalized.

To infer the amount of reduced and oxidized species on the surface at temperatures close to the reduction temperatures observed in the TPR profiles, we performed in situ reductions in the high-pressure cell interfaced to the XPS analysis chamber. In Table S7, the surface atomic percent compositions of the as-prepared catalysts and after in situ reduction inside the HPC at 350 °C are reported. The Ni 2p_3/2_ spectra are shown in Figure 4, whereas the whole Ni 2p + Ce 3d and Fe 2p_3/2_ regions are shown in Figures S5 and S6. As shown in Figure 4 and reported in Table S7, the as-prepared catalysts were completely oxidized and showed very similar profiles for the same composition, irrespective of the synthesis method. The Ni oxides’ composition was about 80% NiO and 20% Ni(OH)_2_ for the monometallic Ni/CeO_2_ samples. The addition of Fe changed the oxides’ composition, and Ni(OH)_2_ reached ca. 30% in Fe_0.1_Ni_0.9_/CeO_2_ catalysts and Fe_0.2_Ni_0.8_/CeO_2_-BM and ca. 44% for Fe_0.2_Ni_0.8_/CeO_2_-IWI. In agreement with the XRD results that showed smaller NiO NPs upon Fe addition for the IWI samples, the (Ni + Fe)/Ce atomic ratio increased with the addition of Fe due to the higher amount of metals’ surface exposed. The Ni/CeO_2_-BM and Fe_0.1_Ni_0.9_/CeO_2_-BM catalysts showed a higher (Ni + Fe)/Ce atomic ratio compared to the IWI counterparts, in agreement with the XRD results as well. This may be explained by the fact that the NiO nanoparticles are smaller for the ball-milled catalysts than for the traditionally prepared catalysts, as inferred from XRD (Table S3). The Fe_0.2_Ni_0.8_/CeO_2_-BM sample showed a lower (Ni + Fe)/Ce ratio compared to the other samples, which can be ascribed to the lower metal loading measured by ICP-OES combined with the different trend observed with XRD where larger NiO NPs were detected for the BM sample than for the IWI. The XPS spectra of the samples after in situ reduction in the HPC at 350 °C are shown in Figure 4. The substitution of Ni with Fe decreased the reducibility of Ni oxide species and led to the presence of residual Ni oxide species. About 2% of Ni(OH)_2_ was present for Ni/CeO_2_-BM, whereas for the Ni/CeO_2_-IWI sample, Ni(OH)_2_ was about 15%. For both Fe_0.1_Ni_0.9_/CeO_2_ samples, the amount of metallic Ni was about 45%. Increasing the amount of Fe samples led to a further decrease in the percentage of metallic Ni to 19% and 28% for Fe_0.2_Ni_0.8_/CeO_2_ IWI and BM, respectively. No shift in binding energy was identified for the metallic Ni peaks. These results are in agreement with the TPR results (Figure 1) that showed a shift to higher temperatures of the reduction temperature maxima for the samples with larger amounts of Fe.

Furthermore, these results show that the BM samples are more easily reduced than the IWI counterparts, probably due to a stronger interaction between ceria and Ni and Fe. Regarding the metal dispersion, the (Ni + Fe)/Ce ratio decreased by an average of 68% compared to the as-prepared state for all samples. This can be related to the sintering of the metallic NPs, as confirmed by TEM analyses (Figure 2 and Figure S1) that showed the presence of very small clusters in the as-prepared catalysts that could agglomerate, lowering the XPS signal. Finally, the Fe/Ni ratio decreased for all the samples during the reduction, showing a reorganization of the NPs’ surface composition exposing more Ni, as expected under reducing conditions.^62^ The (Ni + Fe)/Ce ratios showed the same trends as the respective calcined samples.

### Catalytic Activity and Postreaction Characterization

**Figure 5:**
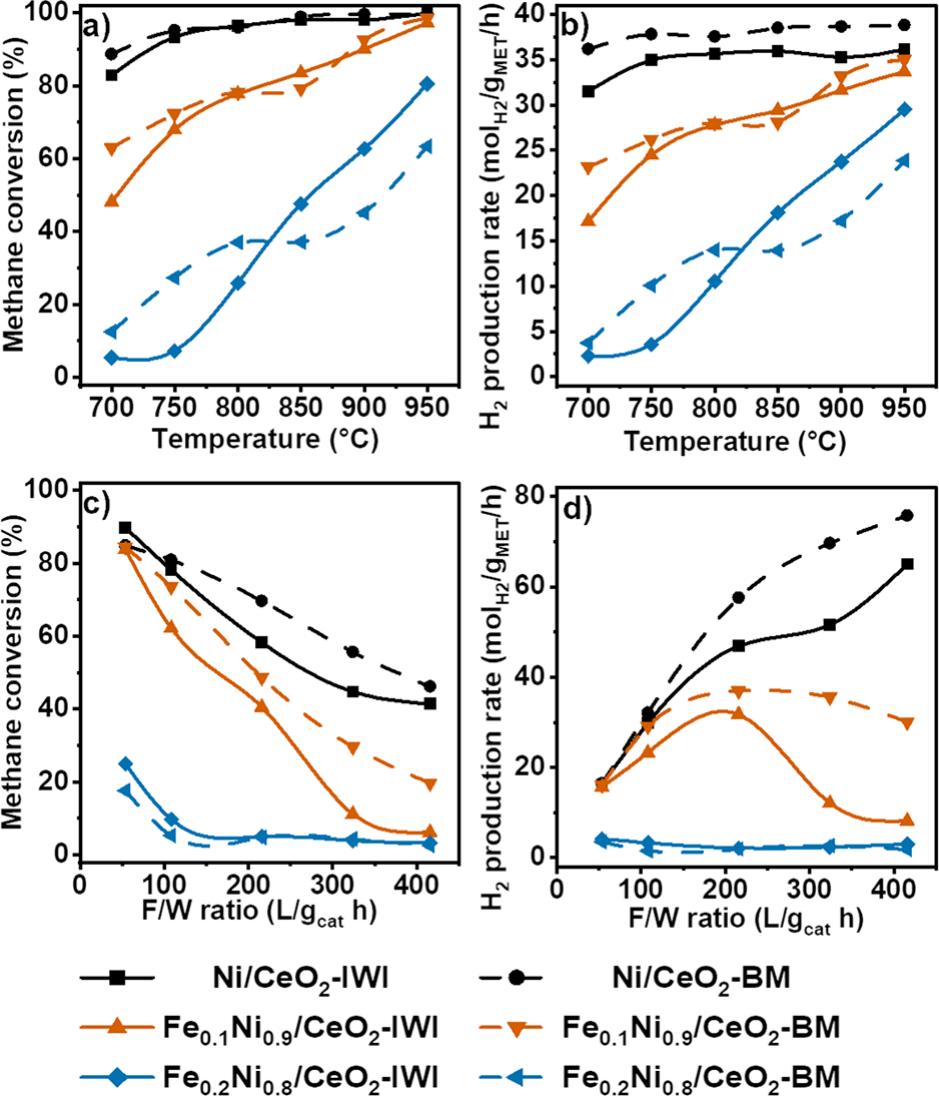
(a) Methane conversion and (b) hydrogen production rates of NiFe/CeO_2_ catalysts at the indicated reaction temperatures (F/W = 108,000 mL g_cat_^–1^ h^–1^). (c) Methane conversion and (d) hydrogen production rates at the indicated F/W ratios (*T* = 700 °C). Values normalized by the metal loading (corresponding to Ni, for monometallic catalysts, or Ni + Fe, for bimetallics, loading) determined by ICP-OES. S/C = 2.

The effect of temperature on the catalytic activity was studied between 700 and 950 °C for a total time on stream of about 5 h. Methane conversion (*X*_CH4_) and hydrogen production rate (*r*_H2_) normalized per gram of metal are shown in Figure 5a,b. Methane conversion followed a decreasing trend with the substitution of Ni with Fe, with the monometallic Ni catalysts showing higher conversion rates than the bimetallic NiFe samples. Importantly, *r*_H2_ was higher for the samples prepared by ball milling, which could be related to the differences in metal–support interaction and particle size discussed above, which showed smaller Ni and NiFe NPs for the BM samples, indicating a higher dispersion of these species (see Table S3). Monometallic catalysts reached full conversion at 850 °C, whereas the samples containing 1% Fe reached about 98% methane conversion at 950 °C. The hydrogen production rate of Fe_0.1_Ni_0.9_/CeO_2_-BM reached similar values to the monometallic samples at 900–950 °C. The Fe_0.2_Ni_0.8_/CeO_2_ catalysts show a distinct behavior, as they are the least active catalysts. In the low-temperature regime (700–800 °C, which is the most interesting temperature regime), Fe_0.2_Ni_0.8_/CeO_2_-BM is more active than the IWI counterpart, whereas at temperatures between 850 and 950 °C, the IWI catalyst shows higher methane conversion. Comparing data points at the same conversion levels, the selectivity to CO_2_ is lower for the samples containing Fe. Nevertheless, this can be because they reach the same conversion rate at higher temperatures, where thermodynamics favors the reverse WGSR.

The same trends were observed when changing the F/W ratio between 54 and 415 L g_cat_^–1^ h^–1^ at 700 °C (Figure 5c,d), with the monometallic catalysts showing higher methane conversion than bimetallic catalysts and the BM samples showing higher *X*_CH4_ compared to the IWI counterparts. The methane conversion decreased for all samples as a result of the lower contact times. The normalized *r*_H2_ increased monotonically for the monometallic samples, whereas for Fe_0.1_Ni_0.9_/CeO_2_ samples, the *r*_H2_ showed a maximum value at 216 L g_cat_^–1^ h^–1^, and after that, the production rate decreased.

Nevertheless, for the milled sample, which showed higher methane conversion, the hydrogen production rate underwent a minor decrease, thus maintaining a higher activity with the increase of reactant flows than the IWI counterpart that experienced a greater deactivation. Figure S8 reports the products’ selectivity of the load experiments. The selectivity toward H_2_ remained constant for the monometallic catalysts and Fe_0.1_Ni_0.9_/CeO_2_-BM, which are the catalysts with the highest activity. For the other samples, the selectivity for H_2_ decreased when increasing the F/W ratio, especially for the Fe_0.2_Ni_0.8_/CeO_2_ catalysts, probably because of the deactivation of the MSR pathway. A comparison of the catalytic activity with results from the literature obtained under similar reaction conditions is reported in Table S8. Our catalysts show similar methane conversion and hydrogen production rates to the ones reported in the open literature.

**Figure 6:**
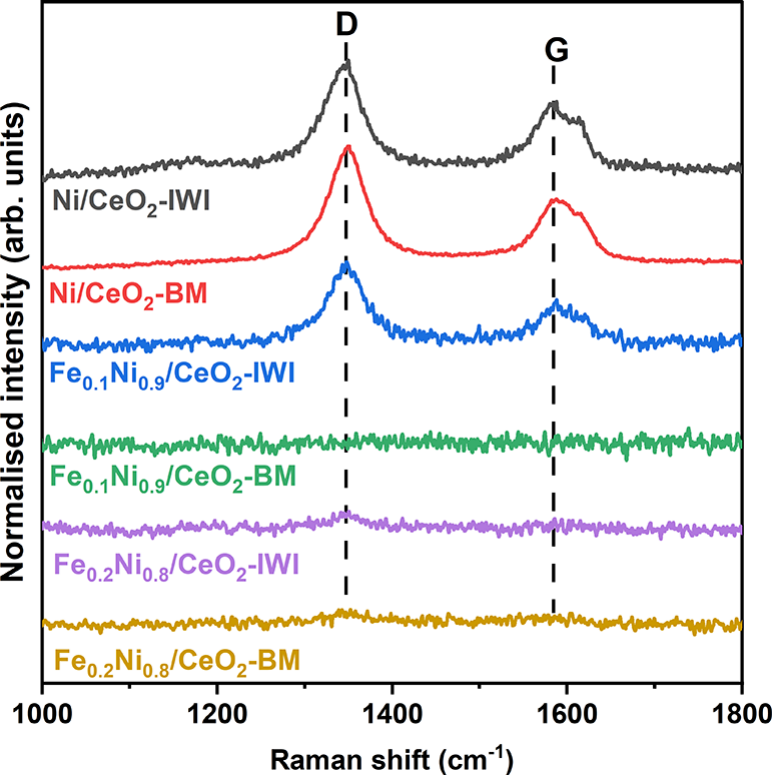
Raman spectra of the carbon region of spent NiFe/CeO_2_ catalysts after the load tests (S/C = 2, *T* = 700 °C, F/W = 54–415 L g_cat_^–1^ h^–1^).

After the load tests with a total duration of ca. 8 h, the spent catalysts were analyzed by Raman spectroscopy. Representative spectra for each catalyst are reported in Figure 6. For monometallic catalysts, carbon signals were clearly detected. The intensity ratio *I*_D_/*I*_G_ between the D-band, associated with amorphous and defective carbon and which can be removed at lower temperatures, and the G-band, associated with crystalline graphitic carbon,^52,63^ was 2.0 for both Ni/CeO_2_ catalysts. For the bimetallic Fe_0.1_Ni_0.9_/CeO_2_-IWI sample, the *I*_D_/*I*_G_ ratio was 2.7, higher compared to the monometallic catalysts probably due to a more oxidizing environment in the presence of Fe that leads to the formation of more defective carbon. In contrast, no carbon could be detected in the BM counterpart. Regarding the Fe_0.2_Ni_0.8_/CeO_2_ catalysts, very low-intensity carbon peaks were detected, likely due to the low catalytic activity achieved by the Fe_0.2_Ni_0.8_/CeO_2_ catalysts.

Thermogravimetric analyses were also performed for the milled catalysts (Figure S9) after the load tests. For the monometallic Ni/CeO_2_-BM sample, after the initial mass loss observed for the three catalysts, a second mass loss event started at ca. 450 °C, which can be ascribed to carbon removal. The total mass loss related to carbon removal for Ni/CeO_2_-BM represented ca. 2 wt % of the total mass, indicating limited carbon deposition. Interestingly, for the milled Fe-containing nanocatalysts, no mass loss related to carbon removal was observed. This result supports our Raman observations.

The surface of the spent catalysts after the reaction at 950 °C was analyzed with ex situ XPS. The atomic ratios of Ni, Fe, and Ce of these catalysts are reported in Table S7, whereas the Ce 3d + Ni 2p high-resolution spectra are shown in Figure S10. For these samples, a detailed chemical analysis of Ni species was not performed because the samples were transferred in air and Ni completely reoxidized. Nevertheless, the total area of Ni 2p_3/2_ peaks was measured to compare the (Ni + Fe)/Ce ratios with those of the in situ reduced samples. After the MSR tests, the surface of the catalysts was richer in Fe, especially for the BM samples, indicating an Fe segregation toward the surface during MSR. Fe segregation is expected under oxidizing environments; thus, this segregation can be led by the presence of hydroxyls on the surface.^62,64^ The (Ni + Fe)/Ce ratio increased with respect to the values obtained after the in situ reduction for the Ni/CeO_2_ and Fe_0.1_Ni_0.9_/CeO_2_ in both IWI and BM catalysts even though they had been exposed to MSR at 950 °C, where sintering occurred (Table S3). The metals/Ce ratio is lower than for the as-prepared catalysts, confirming the results obtained with XRD that indicate that the NPs sinter during reduction and reaction, and it is higher than after reduction, indicating that the sintering observed during reduction is reversible to some extent. Regarding the Fe_0.2_Ni_0.8_/CeO_2_ samples, both showed a lower (Ni + Fe)/Ce ratio (0.30), indicating that the extent of the redispersion for these samples was limited compared to the other sample with lower Fe content. Comparing with the CeO_2_ before reaction, the samples after reaction showed higher Ce^3+^ contribution (see Figure S10), suggesting that the surface of the support was active during the reaction as an oxygen donor.

### NAP-XPS of Fe_0.1_Ni_0.9_/CeO_2_-BM under MSR

**Figure 7:**
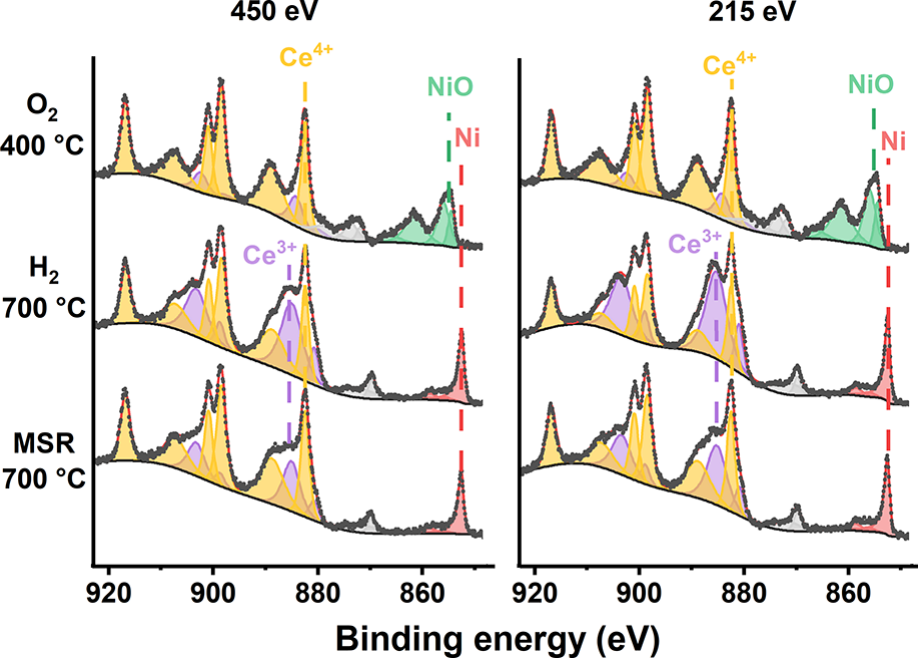
NAP-XPS spectra of Ce 3d and Ni 2p of Fe_0.1_Ni_0.9_/CeO_2_-BM under the indicated gas mixtures and temperatures at 1 mbar for the two sampling depths: 450 and 215 eV, corresponding to the subsurface and surface regions, respectively.

**Figure 8:**
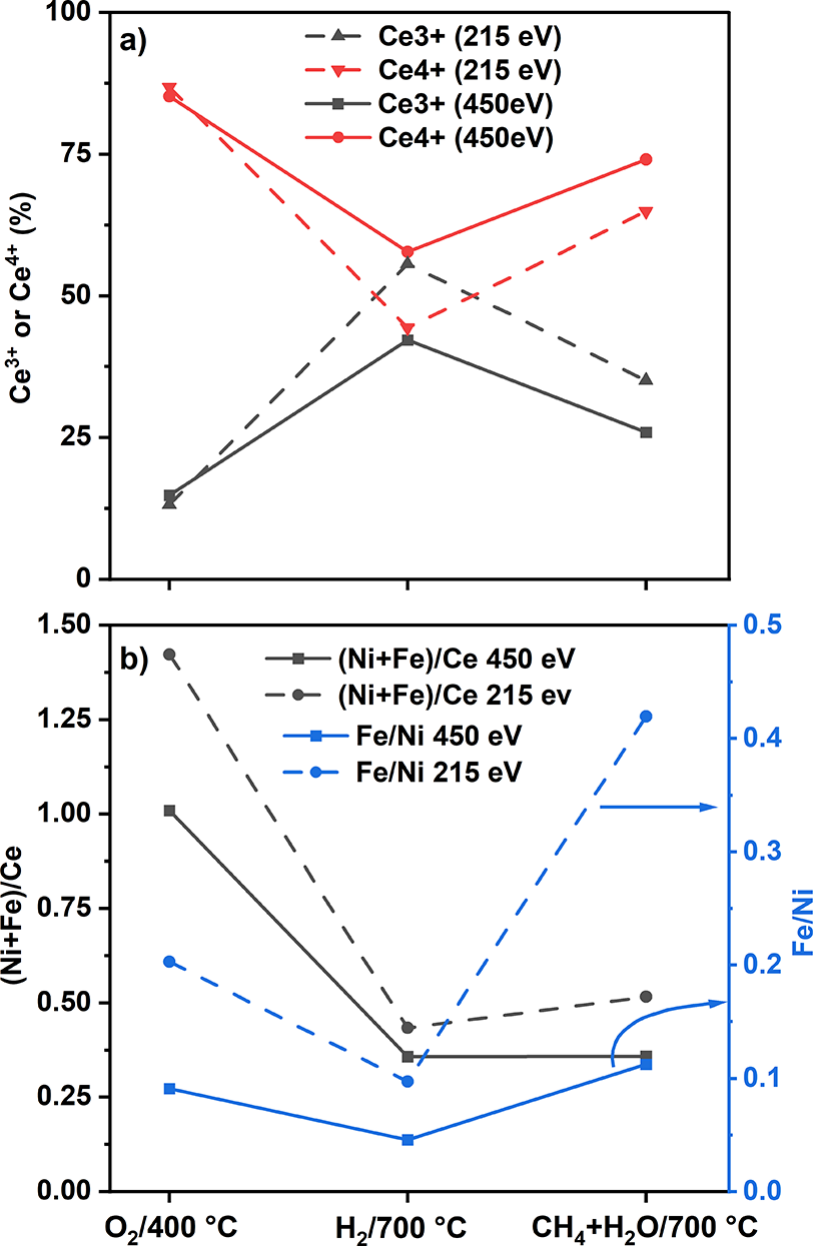
(a) Oxidation state of Ce and (b) (Ni + Fe)/Ce and Fe/Ni atomic ratios of Fe_0.1_Ni_0.9_/CeO_2_-BM surface at 1 mbar of the different gas mixtures and the two photon energies of 215 and 450 eV.

To further investigate the reorganization of Ni and Fe during MSR, the surface of the sample Fe_0.1_Ni_0.9_/CeO_2_-BM was investigated by in situ NAP-XPS.^65^ In Table 1, the surface composition of Fe_0.1_Ni_0.9_/CeO_2_-BM under the atmospheres indicated and for the two photoelectron kinetic energies studied (surface at 215 eV and subsurface at 450 eV, Table S1) are reported, whereas in Figure 7, the NAP-XPS spectra of Ce 3d and Ni 2p are shown. In Figure 8, the oxidation state of cerium during the experiment and the (Ni + Fe)/Ce ratios are plotted together with the Fe/Ni ratios. Under O_2_ at 400 °C, the Ni 2p_3/2_ shape is similar to the one observed under UHV after calcination (Figure 4), with a sharp peak at 854.5 eV and a broader peak at 856.0 eV, compatible with the main peaks of NiO and Ni(OH)_2_. Fe 2p_3/2_ showed a shape compatible with Fe_2_O_3_, with the main peak at 710 eV and a broad satellite at 718 eV (Figure S11).

**Table 1:** Surface Composition of the Sample Fe_0.1_Ni_0.9_/CeO_2_-BM at 1 mbar during Different Gas Treatments Measured by NAP-XPS

treatment	Ce^3+^	Ce^4+^	Ni/Ce	Fe/Ce	Fe/Ni	(Ni + Fe)/Ce
O_2_/400 °C subsurface	14.8%	85.2%	0.93	0.08	0.09	1.01
O_2_/400 °C surface	13.2%	86.8%	1.18	0.24	0.20	1.42
H_2_/700 °C subsurface	42.2%	57.8%	0.34	0.02	0.05	0.36
H_2_/700 °C surface	55.7%	44.3%	0.39	0.04	0.10	0.43
MSR/700 °C subsurface	25.9%	74.1%	0.32	0.04	0.11	0.36
MSR/700 °C surface	35.1%	64.9%	0.36	0.15	0.42	0.52

The (Fe + Ni)/Ce ratios were comparable with the values measured for the same sample under UHV (see Table S7, IMFP ≈ 0.90 nm for Mg X-ray photons). The Fe/Ni ratio was 0.09 at *E*_K_ = 450 eV (IMFP ≈ 0.87 nm) and 0.20 at *E*_K_ = 215 eV (IMFP ≈ 0.57 nm), indicating that the topmost surface layers were richer in Fe compared to the subsurface that showed an Fe/Ni ratio closer to the bulk one (0.11), which is in agreement with the reorganization expected under oxidizing conditions. The oxygen region exhibited two peaks, the first centered at 529.6 eV and the second at 531.2 eV, corresponding to lattice oxygen in the CeO_2_ and to NiO oxides as well as adsorbed oxygen species (Figure S11)^54, 66^; at the same time, two peaks of gaseous O_2_ were visible at 538.5 eV.

During the in situ reduction at 700 °C, Ni and Fe were completely reduced (Figure 7 and Figure S11), and the ratio (Ni + Fe)/Ce decreased to 0.36 and 0.43 at the subsurface and surface regions, respectively, corresponding to a decrease of 67% from the values measured during calcination, similar to that observed after the in situ reduction inside the HPC at 1 bar and 350 °C (Table S7). The Fe/Ni ratio decreased to 0.05 and 0.10 for the two sampling depths. Moreover, the surface of CeO_2_ was reduced, showing a higher Ce^3+^ concentration (Figures 7 and 8). The amount of Ce^3+^ increased from 14.8 and 13.2% obtained under the O_2_ atmosphere to 42.2 and 55.7% for *E*_K_ = 450 and 215 eV, respectively. The O 1s region changed upon ceria reduction, showing two components at 529.6 and 530.6 eV compatible with oxygen in CeO_2_ and CeO_2–*x*_ (Figure S11).^66^ Upon dosing the MSR reaction mixture (CH_4_ + H_2_O, S/C = 2), the production of H_2_ and CO, together with some CO_2_, was observed with the mass spectrometer connected to the analysis chamber, indicating that the catalyst was active for the MSR reaction (Figure S12). No C peaks related to carbon deposition were observed.

During in situ MSR at 700 °C, Ni remained fully reduced, whereas Fe was oxidized with a shape compatible with Fe_3_O_4_ or FeO,^54^ showing a broad peak at 710 eV. Cerium oxide was more reduced than under O_2_ and more oxidized than under H_2_ at the same temperature, with 25.9 and 35.1% of Ce^3+^ for *E*_K_ = 450 and 215 eV, respectively, suggesting that, under MSR, the ceria support participates in the reaction, probably by activating water. The O 1s region showed an isolated peak corresponding to H_2_O vapor at 535 eV, two peaks at 529.6 and 530.7 eV corresponding to oxygen in CeO_2_ and in CeO_2–*x*_, respectively, and a weak peak centered at 531.8 eV that can be attributed to surficial OH^–^ (Figure S11).

The ratio (Ni + Fe)/Ce under MSR increased from 0.43 to 0.52 at 215 eV, mainly as a result of the increase of exposed Fe. The Ni/Ce ratio for both the surface and subsurface regions slightly decreased. However, a strong difference was observed for the Fe/Ce ratio, as a remarkable increase of the Fe/Ce ratio for the surface region was observed (0.04 vs 0.15) when changing from reducing conditions to MSR at the same temperature (700 °C). This resulted in a strong variation of the Fe/Ni ratio upon dosing methane and water. Sampling the subsurface at 450 eV, the Fe/Ni ratio was 0.11, similar to the value of the sample after the calcination treatment, whereas the outermost region (sampled at 215 eV) showed a ratio of Fe/Ni = 0.42. Considering that the signal coming from the subsurface also contains the contribution of the surface, a very strong Fe segregation toward the first surface layers occurred, and Fe oxide was fundamentally located in the topmost layers of the NPs. Iron segregation toward the surface is expected under oxidizing conditions, whereas Fe migration toward the core of the NiFe NPs will occur under a reducing atmosphere,^62^ in agreement with our experiments. The outward trend of Fe observed under MSR conditions at 700 °C (the same temperature as for the reduction step), despite H_2_ and CO being produced on the surface, points to an adsorbate-induced segregation.^67^

## Discussion

Our catalytic performance tests showed that higher normalized H_2_ production rates, as well as a more stable performance (load tests, Figure 5c,d), are attained with the nanocatalysts synthesized by BM, for both mono- and bimetallic formulations, in comparison to the traditionally prepared counterparts, revealing an important influence of the synthesis method on the catalytic activity. By ball milling, smaller NPs were stabilized, and they kept a smaller size during the whole in situ SXRD series (Figure 3f). Furthermore, their reducibility was enhanced, and a larger fraction of metallic Ni was found by XPS after the in situ reduction inside the HPC at 350 °C (Table S7) than for IWI catalysts. Iron addition resulted in lower methane conversions for all the concentrations studied, indicating that Fe addition modified the properties of Ni/CeO_2_ catalysts, resulting in the monometallic Ni/CeO_2_-BM catalyst being the best one. This is in contrast to previous studies, where bimetallic NiFe NPs with an Fe/(Ni + Fe) ratio between 0.1 and 1 supported on Al_2_O_3_ or MgAl_2_O_4_^39^ showed better performances and stability under time-on-stream than the monometallic Ni/Al_2_O_3_ or Ni/MgAl_2_O_4_ counterparts. This reveals the strong influence of the support material in the catalytic behavior. In our work, CeO_2_ was chosen as a support material, which is a basic support with a strong redox behavior,^44^ in contrast to acidic/nonreducible Al_2_O_3_ and MgAl_2_O_4_. Although Fe can improve the long-term stability of NiFe catalysts based on alumina through coke oxidation and CO activation around clusters of FeO_*x*_ segregated on Ni-rich particles, the greater redox activity of ceria outperforms FeO_*x*_ effects, resulting in catalysts with loss of Ni active sites and lowered activity.

On the basis of the microreversibility principle in catalysis, information obtained for the Sabatier reactions (CO_2_ and CO methanation), where NiFe catalysts are widely studied and employed,^40^ can be useful for MSR. The materials employed for the reforming reactions and the methanation reactions are similar, and comparable syntheses and physical–chemical properties have been used. Similarly to what is reported in the literature for steam reforming, bimetallic NiFe catalysts supported on nonreducible supports such as Al_2_O_3_, MgAl_2_O_4_, and SiO_2_ showed improved catalytic activity and stability compared to the monometallic counterparts for CO and CO_2_ methanation thanks to the redox properties of Fe (promotion of coke oxidation and CO_2_ activation^40^). On the contrary, when the support was a redox material such as CeO_2_, the catalytic activity was lowered upon Fe addition because the addition of Fe changed the interaction between the adsorbate molecules and the active Ni species and the number of active Ni sites were lowered as a result of the presence of segregated FeO_x._^68−70^ These results are in line with our observations for the MSR reaction. In fact, our NAP-XPS studies revealed a strong Fe migration toward the surface probably accompanied with some segregation of Fe oxides when changing from a hydrogen atmosphere at 700 °C to MSR conditions at the same temperature.

Our physicochemical characterization showed that Fe addition led to the formation of smaller NPs, as previously reported, and from our synchrotron SXRD, we showed that this trend is kept under the high temperatures required to activate Ni-based catalysts and to run the MSR reaction. Interestingly, our SXRD measurements showed the formation of metallic Ni NPs under reaction and of NiFe alloy NPs, demonstrating the inclusion of Fe in the Ni lattice, and no oxide species were detected. Nevertheless, our surface-sensitive NAP-XPS measurements showed the presence of Fe oxide species on the outmost layers of the NiFe-BM catalyst, which indicates that the FeO_*x*_ NPs formed are very small, escaping XRD detection, and that they are strongly located on the surface. In our nanomaterials, Fe addition had a notable effect on carbon formation as well. The carbonaceous species detected on the surface of Fe_0.1_Ni_0.9_/CeO_2_-IWI, as determined by Raman spectroscopy (Figure 6), are more defective than those found on monometallic Ni/CeO_2_-IWI and -BM. Remarkably, no C was found on Fe_0.1_Ni_0.9_/CeO_2_-BM. This indicates that despite the presence of a reducible support like ceria, Fe further promoted the oxidation of C, and its addition led to the formation of less graphitic carbon. This can have a direct impact on the long-term performance of MSR catalysts, overcoming one of the main drawbacks of the Ni catalysts supported on alumina and magnesium aluminate spinels, which are the most widely used supports.^3,6^ Therefore, NiFe/CeO_2_ bimetallic nanocatalysts are suitable candidates to enhance the stability of Ni-based catalysts.

## Conclusions

The addition of Fe to Ni/CeO_2_ catalysts for methane steam reforming led to a change in the properties of Ni/CeO_2_ nanomaterials and consequently to their catalytic activity. As expected, the addition of Fe led to a higher dispersion of the NiFe nanoparticles compared to the monometallic samples, as observed by in situ synchrotron XRD and XPS. Nanocatalysts prepared by mechanochemistry showed higher reducibility as well as higher H_2_ production rates compared to impregnated ones due to smaller Ni or NiFe nanoparticles and stronger metal–support interactions. Monometallic samples showed the highest catalytic activity compared to bimetallic nanocatalysts, despite the latter having higher metal dispersion on the surface. By studying the surface of bimetallic NiFe/CeO_2_ prepared by mechanochemistry by in situ NAP-XPS during methane steam reforming, we observed strong Fe segregation toward the surface, which could be the reason behind the lower catalytic activity. Nevertheless, the same nanomaterial proved to be more robust than the impregnated counterpart because higher coke resistance compared to monometallic catalysts, as evidenced by Raman spectroscopy after reaction in harsh conditions, was obtained.

## CRediT Authorship Contributions

Andrea Braga: Investigation, Methodology, Formal analysis, Writing – original draft; Marina Armengol-Profitós: Investigation, Writing – review & editing; Laia Pascua-Solé: Investigation, Writing – review & editing; Xavier Vendrell: Investigation, Writing – review & editing; Lluís Soler: Investigation, Writing – review & editing; Isabel Serrano: Investigation, Writing – review & editing; Ignacio J. Villar-Garcia: Investigation, Writing – review & editing; Virginia Pérez-Dieste: Investigation, Writing – review & editing; Núria J. Divins: Conceptualization, Investigation, Methodology, Supervision, Validation, Writing – original draft, review & editing; Jordi Llorca: Conceptualization, Methodology, Supervision, Validation, Writing – review & editing, Funding acquisition.

## Supporting Information Available

The inelastic mean free path of the photoelectrons generated during NAP-XPS experiments; Ni and Fe concentrations determined by ICP-OES; parameters obtained from the Rietveld analyses; hydrogen yields reached during the catalytic experiments; surface composition of the nanocatalysts as-prepared, after in situ reduction, and post reaction; catalytic performance comparison of the investigated catalysts with data reported in the open literature (Tables S1–S8); TEM images; in situ diffractograms; XPS, NAP-XPS, and in situ recorded spectra as well as the corresponding MS signals of the NAP-XPS measurements; product selectivity of the catalytic activity tests; and TGA analyses of the ball-milled samples (Figures S1–S12) (PDF)
