# Supplementary material for: Bimetallic NiFe Nanoparticles Supported on CeO2 as Catalysts for Methane Steam Reforming
Source: ACS Appl Nano Mater. 2023 May 2;6(9):7173–85. doi: 10.1021/acsanm.3c00104 (PMC10186329; doi:10.1021/acsanm.3c00104)
Supplement: Supplementary file 1 — an3c00104_si_001.pdf [file an3c00104_si_001.pdf]

Supporting Information

# Bimetallic NiFe Nanoparticles Supported on $CeO_2$ as Catalysts for Methane Steam Reforming

Andrea Braga,[a,b,c] Marina Armengol-Profitós,[a,b,c] Laia Pascua-Solé,[a,c] Xavier Vendrell,[a,c] Lluís Soler,[a,b,c] Isabel Serrano,[a] Ignacio J. Villar-Garcia,[d] Virginia Pérez-Dieste,[d] Núria J. Divins*[a,b,c,] Jordi Llorca*,[a,b,c]

[a] Institute of Energy Technologies, Universitat Politècnica de Catalunya, EEBE, Eduard Maristany 10–14, 08019 Barcelona, Spain.
[b] Department of Chemical Engineering, Universitat Politècnica de Catalunya, EEBE, Eduard Maristany 10–14, 08019 Barcelona, Spain.
[c] Barcelona Research Center in Multiscale Science and Engineering, Universitat Politècnica de Catalunya, EEBE, Eduard Maristany 10–14, 08019 Barcelona, Spain.
[d] ALBA Synchrotron Light Source, Carrer de la Llum 2-26, 08290 Cerdanyola del Vallès, Barcelona, Spain.
*Corresponding authors: jordi.llorca@upc.edu, nuria.jimenez.divins@upc.edu

**Table S1**. Inelastic mean free path values for Ce, Ni, and Fe at different electron kinetic energies, and the corresponding photon energies used for the high-resolution NAP-XPS measurements.

| Region | IMFP - 215 eV (nm) | Photon energy (eV) | IMFP - 450 eV (nm) | Photon energy (eV) |
|---|---|---|---|---|
| **Ce 3d+Ni 2p** | 0.7 | 1100 | 1.1 | 1335 |
| **Ni 2p** | 0.6 | 1100 | 0.8 | 1335 |
| **Fe 2p** | 0.6 | 936 | 0.9 | 1171 |

**Table S2**. Fe and Ni weight concentrations as measured by ICP-OES.

| Sample | Ni (%) | Fe (%) | Fe/Ni | Total metal loading (%) |
|---|---|---|---|---|
| **$Ni/CeO_2$-IWI** | 8.4 | 0.0 | 0.00 | 8.4 |
| **$Ni/CeO_2$-BM** | 8.0 | 0.0 | 0.00 | 8.0 |
| **$Fe_{0.1}Ni_{0.9}CeO_2$-IWI** | 7.7 | 0.9 | 0.12 | 8.5 |
| **$Fe_{0.1}Ni_{0.9}CeO_2$-BM** | 7.5 | 0.8 | 0.11 | 8.4 |
| **$Fe_{0.2}Ni_{0.8}CeO_2$-IWI** | 7.1 | 1.8 | 0.25 | 8.9 |
| **$Fe_{0.2}Ni_{0.8}CeO_2$-BM** | 6.2 | 1.6 | 0.26 | 7.8 |

**Table S3**. Crystallite size of NiO, Ni and NiFe nanoparticles at different conditions and temperatures, obtained from Rietveld refinements.

| Sample | 25 °C $H_2$ NiO (nm) | 700 °C $H_2$ Ni/NiFe (nm) | 700 °C MSR Ni/NiFe (nm) | 800 °C MSR Ni/NiFe (nm) | 950 °C MSR Ni/NiFe (nm) |
|---|---|---|---|---|---|
| **$Ni/CeO_2$-IWI** | 23.8 | 26.0 | 26.2 | 28.1 | 36.9 |
| **$Ni/CeO_2$-BM** | 19.7 | 20.9 | 22.1 | 23.9 | 32.3 |
| **$Fe_{0.1}Ni_{0.9}/CeO_2$-IWI** | 16.7 | 17.7 | 18.9 | 21.2 | 30.8 |
| **$Fe_{0.1}Ni_{0.9}/CeO_2$-BM** | 12.0 | 14.5 | 16.1 | 18.9 | 28.4 |
| **$Fe_{0.2}Ni_{0.8}/CeO_2$-IWI** | 7.8 | 25.6 | - | - | - |
| **$Fe_{0.2}Ni_{0.8}/CeO_2$-BM** | 10.1 | 12.8 | 13.6 | 16.4 | 27.7 |

**Table S4**. NiO, Ni and NiFe lattice parameters after 30' at different conditions and temperatures, calculated from whole pattern Rietveld refinements.

| Sample | 25 °C/$H_2$ NiO (Å) | 700 °C/$H_2$ Ni/NiFe (Å) | 700 °C/MSR Ni/NiFe (Å) | 800 °C/MSR Ni/NiFe (Å) | 950 °C/MSR Ni/NiFe (Å) |
|---|---|---|---|---|---|
| **Ni/$CeO_2$-IWI** | 4.185 | 3.564 | 3.564 | 3.569 | 3.577 |
| **Ni/$CeO_2$-BM** | 4.186 | 3.565 | 3.565 | 3.570 | 3.578 |
| **$Fe_{0.1}Ni_{0.9}CeO_2$-IWI** | 4.187 | 3.574 | 3.574 | 3.580 | 3.589 |
| **$Fe_{0.1}Ni_{0.9}CeO_2$-BM** | 4.185 | 3.571 | 3.571 | 3.577 | 3.585 |
| **$Fe_{0.2}Ni_{0.8}CeO_2$-IWI** | 4.190 | 3.586 | - | - | - |
| **$Fe_{0.2}Ni_{0.8}CeO_2$-BM** | 4.188 | 3.585 | 3.585 | 3.591 | 3.599 |

**Table S5**. $CeO_2$ lattice parameters (Å) at different conditions and temperatures, calculated from whole pattern Rietveld refinements.

| Sample | 25 °C/$H_2$ | 700 °C/$H_2$ | 700 °C/MSR | 800 °C/MSR | 950 °C/MSR |
|---|---|---|---|---|---|
| **Ni/$CeO_2$-IWI** | 5.417 | 5.459 | 5.459 | 5.466 | 5.480 |
| **Ni/$CeO_2$-BM** | 5.419 | 5.461 | 5.460 | 5.467 | 5.481 |
| **$Fe_{0.1}Ni_{0.9}CeO_2$-IWI** | 5.418 | 5.460 | 5.460 | 5.467 | 5.484 |
| **$Fe_{0.1}Ni_{0.9}CeO_2$-BM** | 5.415 | 5.457 | 5.457 | 5.464 | 5.480 |
| **$Fe_{0.2}Ni_{0.8}CeO_2$-IWI** | 5.420 | 5.461 | - | - | - |
| **$Fe_{0.2}Ni_{0.8}CeO_2$-BM** | 5.418 | 5.461 | 5.462 | 5.469 | 5.484 |

**Table S6**. Hydrogen yields of the FeNi/$CeO_2$ catalysts at different reaction temperatures (F/W = 108,000 mL $g_{cat}^{-1}$ $h^{-1}$) and at different flow-to-weight ratios (T = 700 °C).

| Temperature (°C) | Ni/CeO2-IWI | Ni/$CeO_2$-BM | $Fe_{0.1}Ni_{0.9}CeO_2$-IWI | $Fe_{0.1}Ni_{0.9}CeO_2$-BM | $Fe_{0.2}Ni_{0.8}CeO_2$-IWI | $Fe_{0.2}Ni_{0.8}CeO_2$-BM |
|---|---|---|---|---|---|---|
| 700 | 2.8 | 2.9 | 1.5 | 2.1 | 0.2 | 0.3 |
| 750 | 3.1 | 3.0 | 2.2 | 2.3 | 0.3 | 0.8 |
| 800 | 3.2 | 3.1 | 2.5 | 2.5 | 1.0 | 1.2 |
| 850 | 3.2 | 3.1 | 2.6 | 2.5 | 1.7 | 1.1 |
| 900 | 3.1 | 3.1 | 2.8 | 2.9 | 2.2 | 1.4 |
| 950 | 3.2 | 3.1 | 3.0 | 3.1 | 2.8 | 2.0 |
| **F/W (mL $g_{cat}^{-1}$ $h^{-1}$)** | | | | | | |
| 54000 | 2.8 | 2.7 | 2.7 | 2.8 | 0.8 | 0.6 |
| 108000 | 2.6 | 2.6 | 2.0 | 2.5 | 0.3 | 0.1 |
| 216000 | 2.0 | 2.3 | 1.4 | 1.6 | 0.10 | 0.07 |
| 324000 | 1.5 | 1.9 | 0.3 | 1.0 | 0.07 | 0.07 |
| 415800 | 1.5 | 1.6 | 0.2 | 0.7 | 0.07 | 0.04 |

**Table S7**. Composition of the surface of the NiFe/$CeO_2$ samples as-prepared, after an in situ reduction in the HPC at 350 °C, and after MSR reaction at 950 °C as inferred from UHV-XPS measurements.

| Sample | Treatment | Ni species (%) | | | Ni/Ce | Fe/Ce | Fe/Ni | (Ni+Fe)/Ce |
|---|---|---|---|---|---|---|---|---|
| | | Ni metal | NiO | $Ni(OH)_2$ | | | | |
| **$Ni/CeO_2$-IWI** | As-prepared | 0.0 | 79.4 | 20.6 | 0.92 | - | - | 0.92 |
| | Reduced | 85.0 | 0.0 | 15.0 | 0.29 | - | - | 0.29 |
| | Post reaction | - | - | - | 0.60 | - | - | 0.60 |
| **$Fe_{0.1}Ni_{0.9}/CeO_2$-IWI** | As-prepared | 0.0 | 68.0 | 32.0 | 1.05 | 0.14 | 0.13 | 1.19 |
| | Reduced | 46.0 | 18.5 | 35.5 | 0.35 | 0.02 | 0.06 | 0.37 |
| | Post reaction | - | - | - | 0.86 | 0.06 | 0.07 | 0.92 |
| **$Fe_{0.2}Ni_{0.8}/CeO_2$-IWI** | As-prepared | 0.0 | 56.5 | 43.5 | 1.13 | 0.21 | 0.18 | 1.33 |
| | Reduced | 18.6 | 47.7 | 33.7 | 0.41 | 0.03 | 0.07 | 0.43 |
| | Post reaction | - | - | - | 0.25 | 0.04 | 0.16 | 0.30 |
| **$Ni/CeO_2$-BM** | As-prepared | 0.0 | 84.5 | 15.5 | 1.43 | - | - | 1.43 |
| | Reduced | 97.6 | 0.0 | 2.4 | 0.55 | - | - | 0.55 |
| | Post reaction | - | - | - | 0.74 | - | - | 0.74 |
| **$Fe_{0.1}Ni_{0.9}/CeO_2$-BM** | As-prepared | 0.0 | 71.9 | 28.1 | 1.25 | 0.08 | 0.07 | 1.34 |
| | Reduced | 43.5 | 25.9 | 30.6 | 0.37 | 0.00 | 0.01 | 0.37 |
| | Post reaction | - | - | - | 0.65 | 0.22 | 0.33 | 0.87 |
| **$Fe_{0.2}Ni_{0.8}/CeO_2$-BM** | As-prepared | 0.0 | 74.5 | 25.5 | 0.64 | 0.11 | 0.18 | 0.75 |
| | Reduced | 28.2 | 46.8 | 25.0 | 0.23 | 0.01 | 0.06 | 0.24 |
| | Post reaction | - | - | - | 0.24 | 0.06 | 0.26 | 0.30 |

**Table S8**. Comparison of the results obtained in this work with results from the literature with similar testing conditions.

| Catalyst | %Ni (wt.%) | Tp (°C) | S/C | F/W (mL $g_{cat}^{-1}$ $h^{-1}$) | Conversion (%) | $H_2$ production rate ($H_2$ mol $g_{cat}^{-1}$ $h^{-1}$) [$H_2$ mol $g_{Ni}^{-1}$ $h^{-1}$] | $CH_4$ conversion rate ($CH_4$ mol $g_{cat}^{-1}$ $h^{-1}$) [$CH_4$ mol $g_{Ni}^{-1}$ $h^{-1}$] | Ref. |
|---|---|---|---|---|---|---|---|---|
| **$Ni/CeO_2$, ball milling** | 8% | 700 | 2 | 108 000 | 88% | 2.7 [32.9] | 0.8 [8.9] | This work |
| **$Ni/CeO_2$, ball milling** | 8% | 700 | 2 | 415 000 | 46% | 6.1 [66.6] | 1.6 [19.2] | This work |
| **$Fe_{0.1}Ni_{0.9}/CeO_2$, ball milling** | 7.5% | 700 | 2 | 108 000 | 62% | 2.0 [24.6] | 0.6 [10.6] | This work |
| **$Fe_{0.1}Ni_{0.9}/CeO_2$, ball milling** | 7.5% | 700 | 2 | 415 000 | 20% | 2.5 [27.8] | 0.7 [8.2] | This work |
| **$Ni/CeO_2$, Impregnation** | 5% | 700 | 2 | 48 000 | 85% | - | 0.6 [12.0] | [1] |
| **$Ni/Y_2Zr_2O_7$, glycine-nitrate combustion** | 10% | 700 | 2 | 72 000 | 85% | - | 0.9 [9.1] | [2] |
| **$Ni/CeO_2/Al_2O_3$, Spray deposition** | 50-100 mol% | 700 | 2 | 156 000 | 94% | - | 0.5 | [3] |
| **$Ni/Al_2O_3$, wet combustion synthesis** | 33% | 650 | 2 | 270 000 | - | 19.6 [59.4] | 3.1 [9.4] | [4] |
| **Ni(M)/MgAlOx, M = Pt, Rh, Pd, coprecipitation** | 13.5% | 700 | 2 | 210 000 | 94% | - | 2.5 [18.6] | [5] |
| **$NiRu/MgAl_2O_4$, impregnation** | 12% | 700 | 3 | 250 000 | 80% | - | 1.8 [14.9] | [6] |
| **$NiPt/MgAl_2O_4$, Impregnation** | 15% | 600 | 5 | 525 000 | 65% | - | 2.2 [14.52] | [7] |

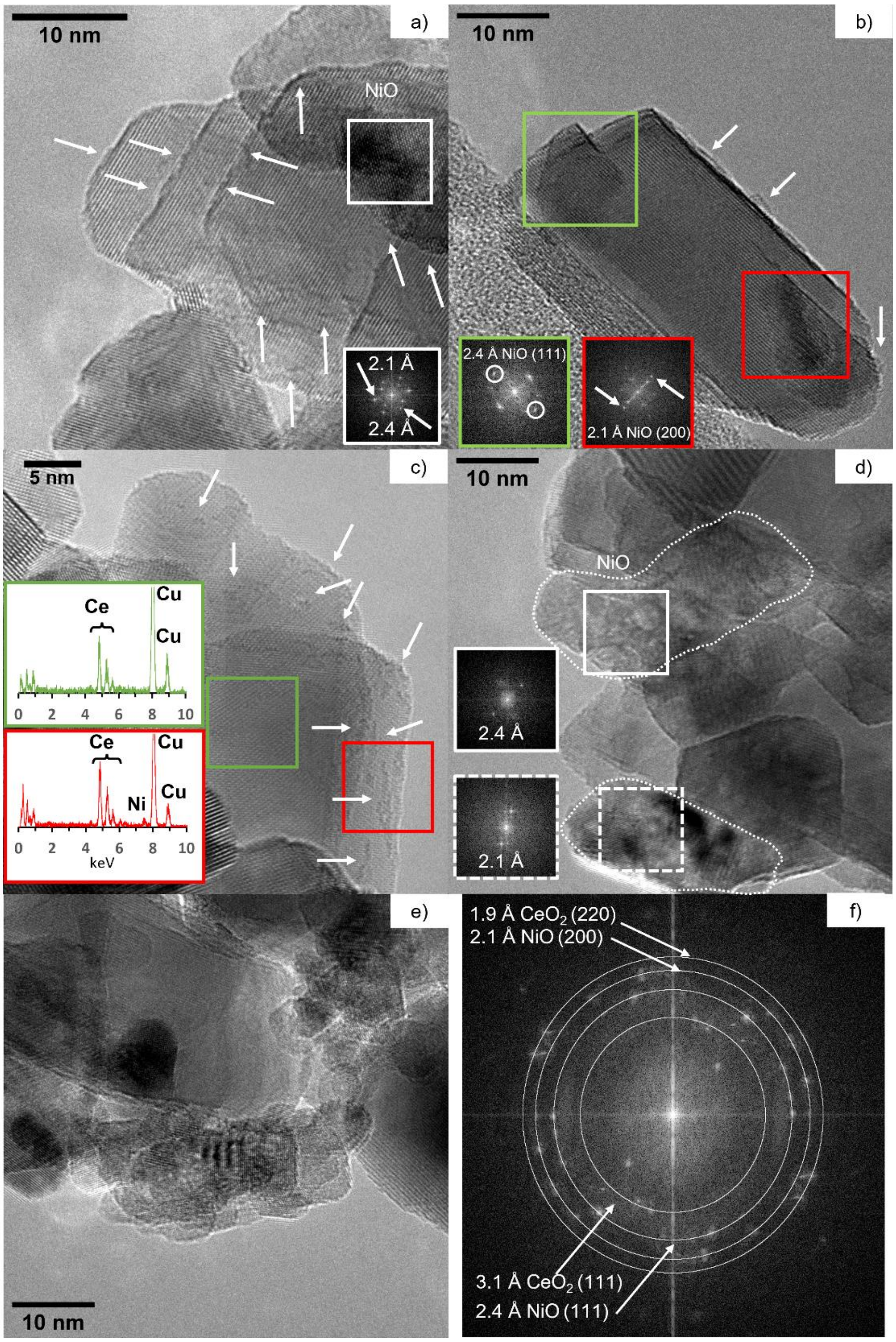


**Figure S1**. HR-TEM of a-b) Ni/$CeO_2$-BM, c-d-e) $Fe_{0.1}Ni_{0.9}/CeO_2$-BM and f) the Fourier transform of e) displaying the spots from $CeO_2$ and NiO crystallographic planes.

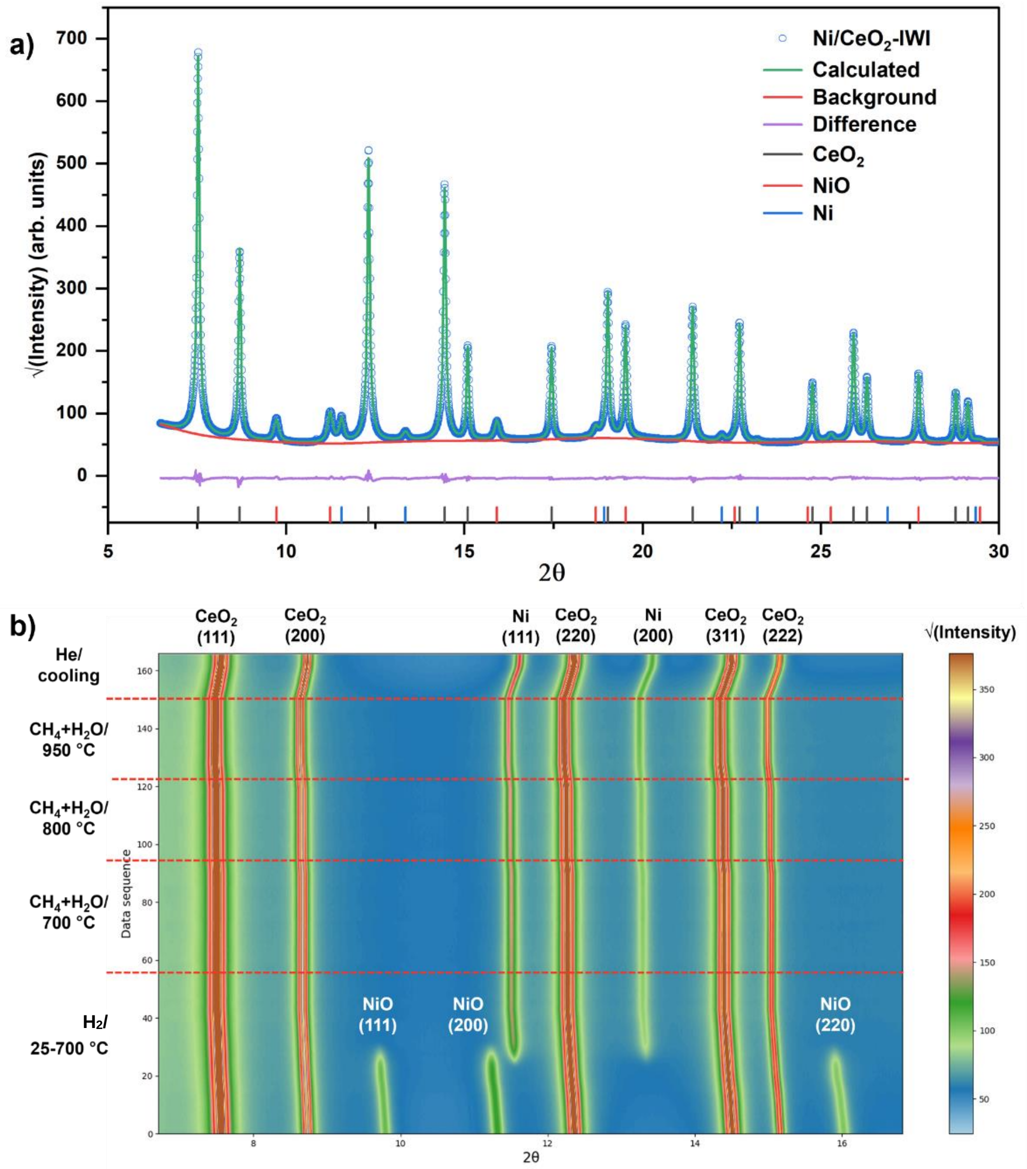


**Figure S2.** Representative figures for the Rietveld refinement analysis used to estimate the NiO, Ni and NiFe nanoparticles size and the lattice parameters shown in Fig. S3-4. In a) it is shown a portion of the diffractogram of the sample Ni/$CeO_2$-BM at 325 °C under $H_2$/Ar and the Rietveld refinement performed in GSAS-II using $CeO_2$, NiO and Ni cubic cells as fitting phases; in b) a detail of the whole in situ SXRD experiment, from room temperature under reducing atmosphere up to 950 °C in MSR reaction mixture is reported, showing the reduction of NiO to Ni and the thermal expansion of the sample at different temperatures. $\lambda$ = 0.413 Å.

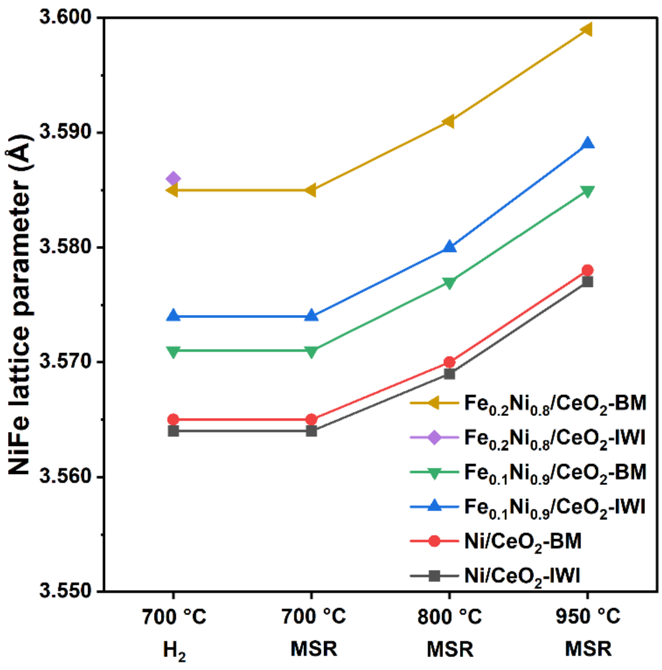


**Figure S3**. Ni and NiFe lattice parameters at different conditions and temperatures calculated from Rietveld refinements.

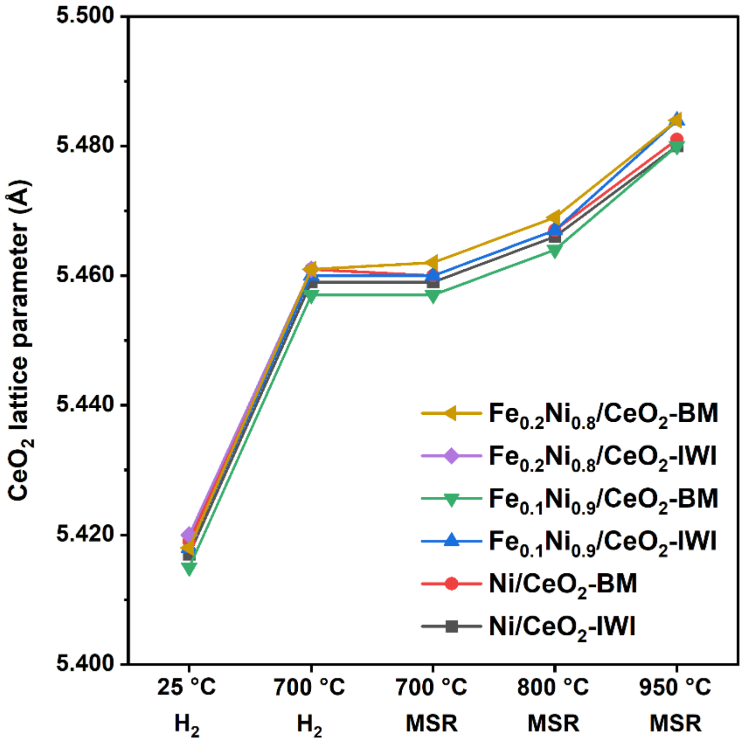


**Figure S4**. $CeO_2$ lattice parameter at different conditions and temperatures calculated from Rietveld refinements.

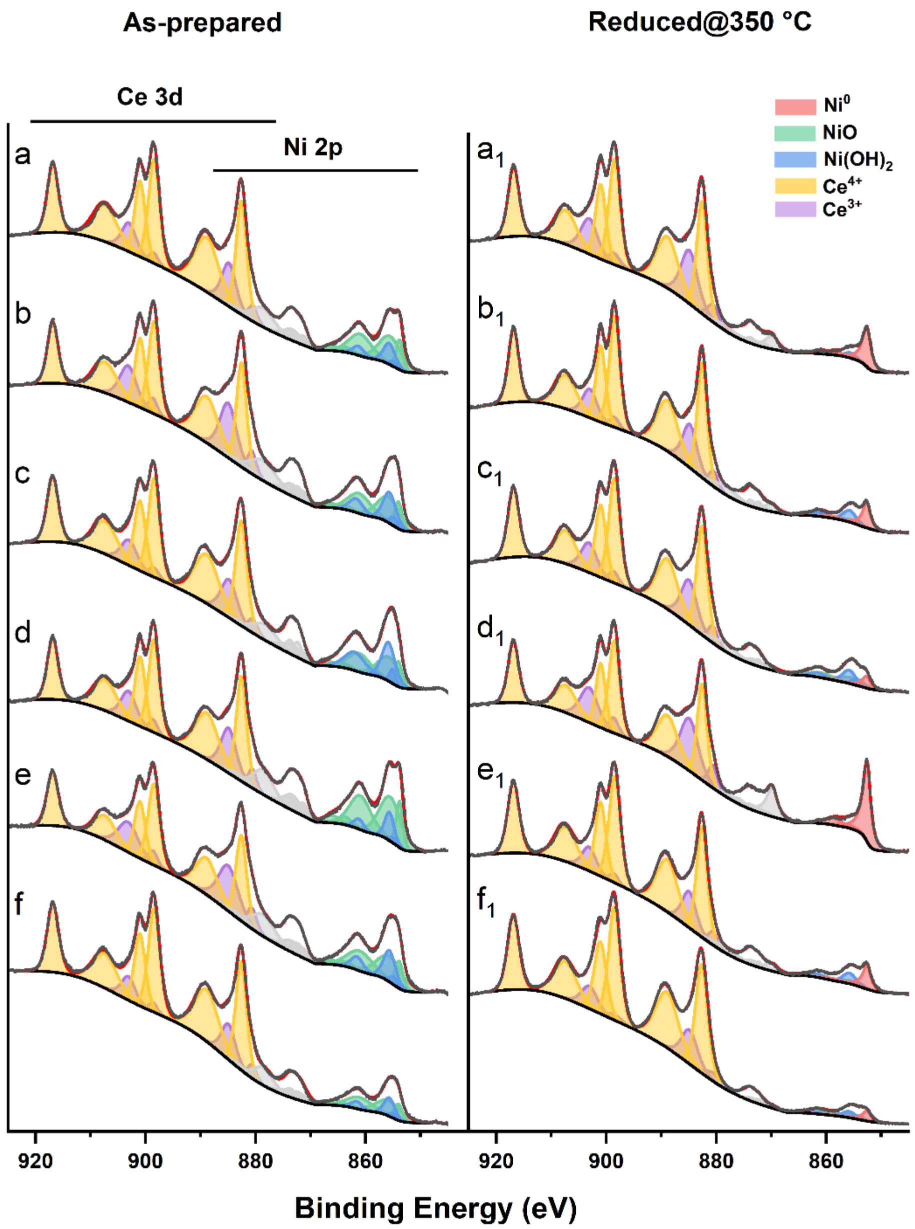


**Figure S5**. Ni 2p + Ce 3d XPS spectra of as-prepared catalysts and after in situ reduction at 350 °C for 30 min under 20 mL min$^{-1}$ of 10% $H_2$/Ar. Spectra are normalized. a-a$_1$) Ni/$CeO_2$-IWI, b-b$_1$) $Fe_{0.1}Ni_{0.9}$/$CeO_2$-IWI, c-c$_1$) $Fe_{0.2}Ni_{0.8}$/$CeO_2$-IWI, d-d$_1$) Ni/$CeO_2$-BM, e-e$_1$) $Fe_{0.1}Ni_{0.9}$/$CeO_2$-BM, f-f$_1$) $Fe_{0.2}Ni_{0.8}$/$CeO_2$-BM.

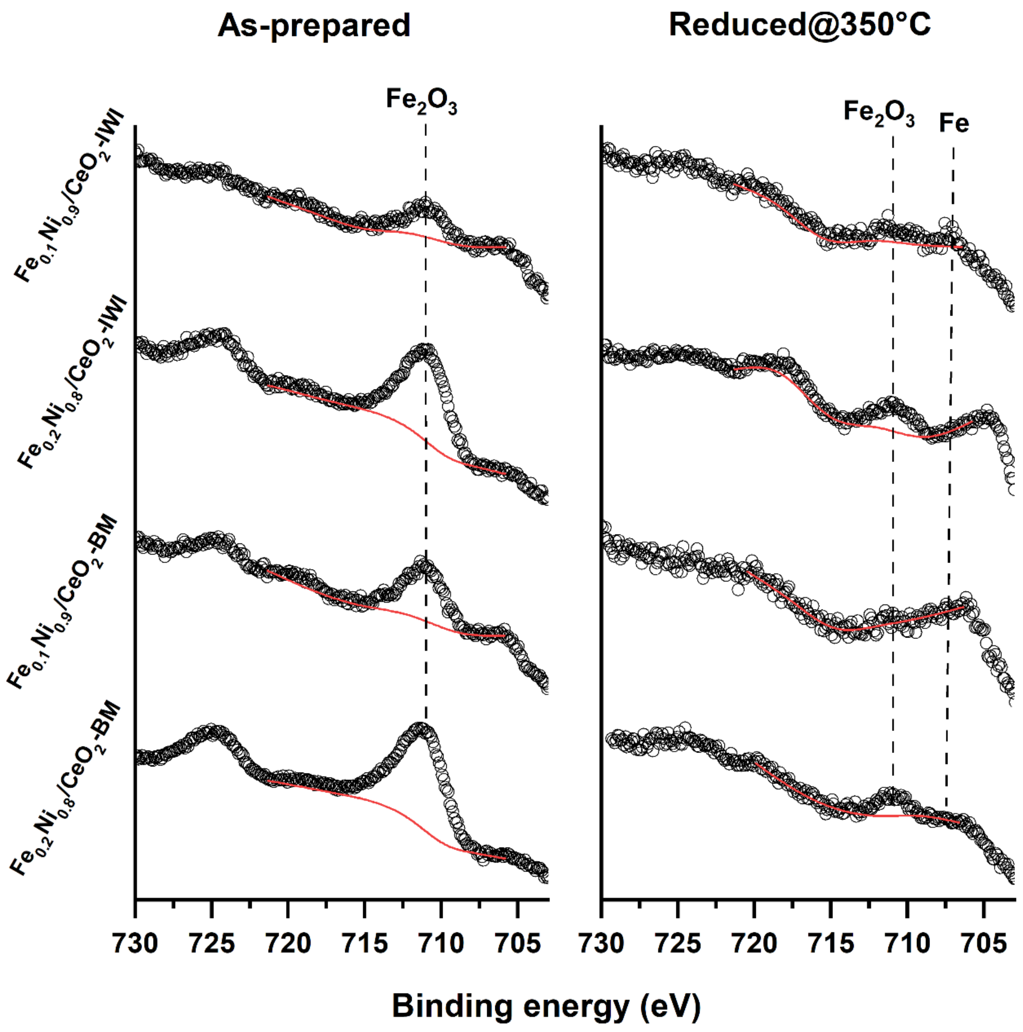


**Figure S6.** Fe $2p_{3/2}$ XPS spectra of as-prepared catalysts and after in situ reduction at 350 °C for 30 min under 20 mL $min^{-1}$ of 10% $H_2$/Ar. Spectra are normalized.

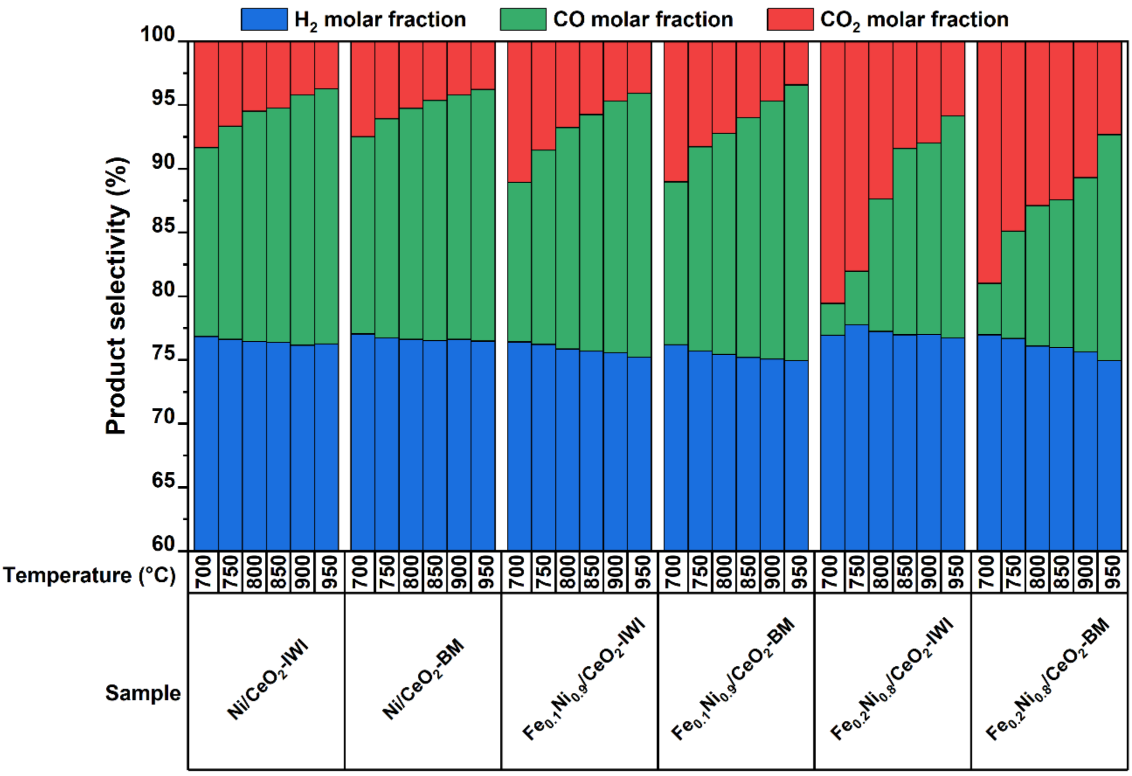


**Figure S7**. Products selectivity of NiFe/$CeO_2$ nanocatalysts at different temperature steps. S/C = 2, F/W = 108 000 mL $g_{cat}^{-1}$ $h^{-1}$.

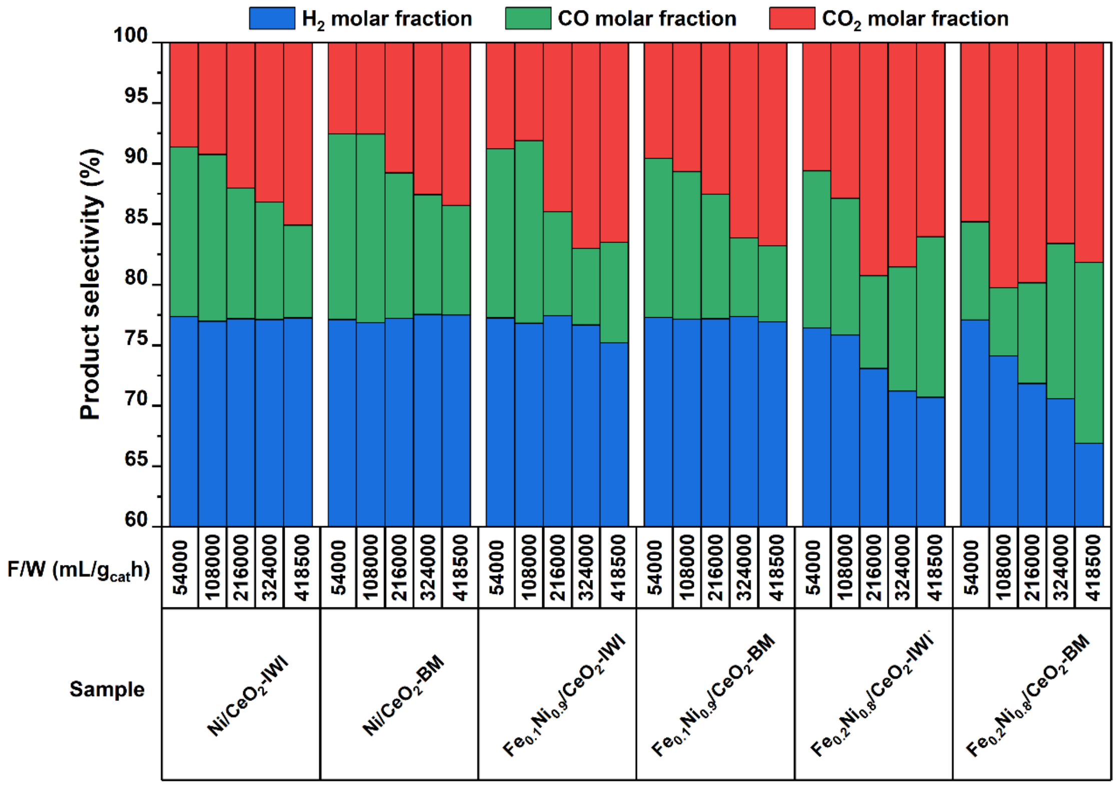


**Figure S8**. Products selectivity of NiFe/$CeO_2$ nanocatalysts at different reactant flows. S/C = 2, T = 700 °C.

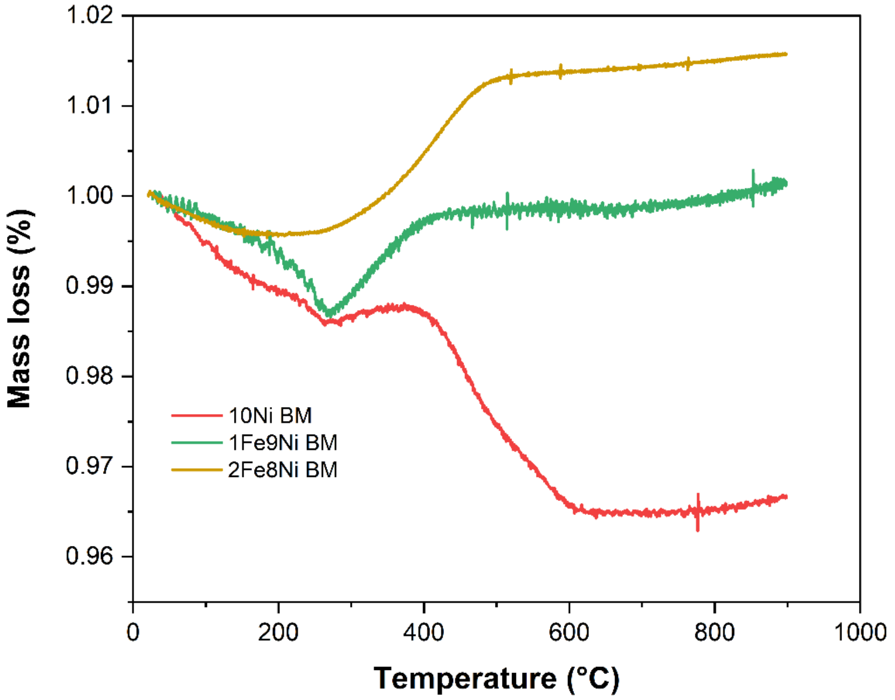


**Figure S9**. Thermogravimetric analyses of the NiFe/$CeO_2$ nanocatalysts prepared by ball milling.

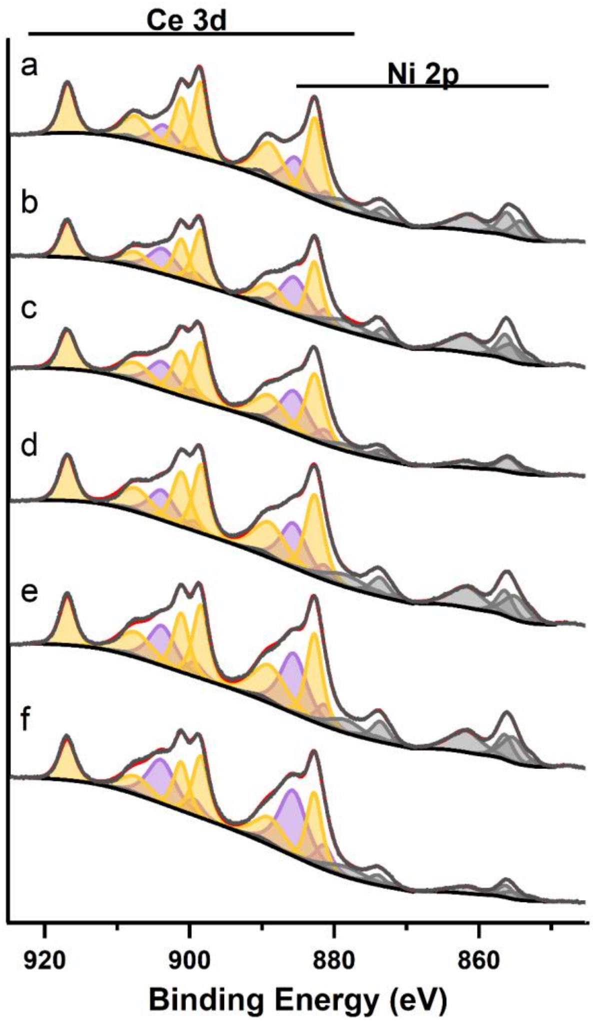


**Figure S10.** XPS spectra of Ni 2p and Ce 3d region of the NiFe/$CeO_2$ nanocatalysts after the reaction temperature steps. Spectra are normalized. a) Ni/$CeO_2$-IWI, b) $Fe_{0.1}Ni_{0.9}$/$CeO_2$-IWI, c) $Fe_{0.2}Ni_{0.8}$/$CeO_2$-IWI, d) Ni/$CeO_2$-BM, e) $Fe_{0.1}Ni_{0.9}$/$CeO_2$-BM, f) $Fe_{0.2}Ni_{0.8}$/$CeO_2$-BM.

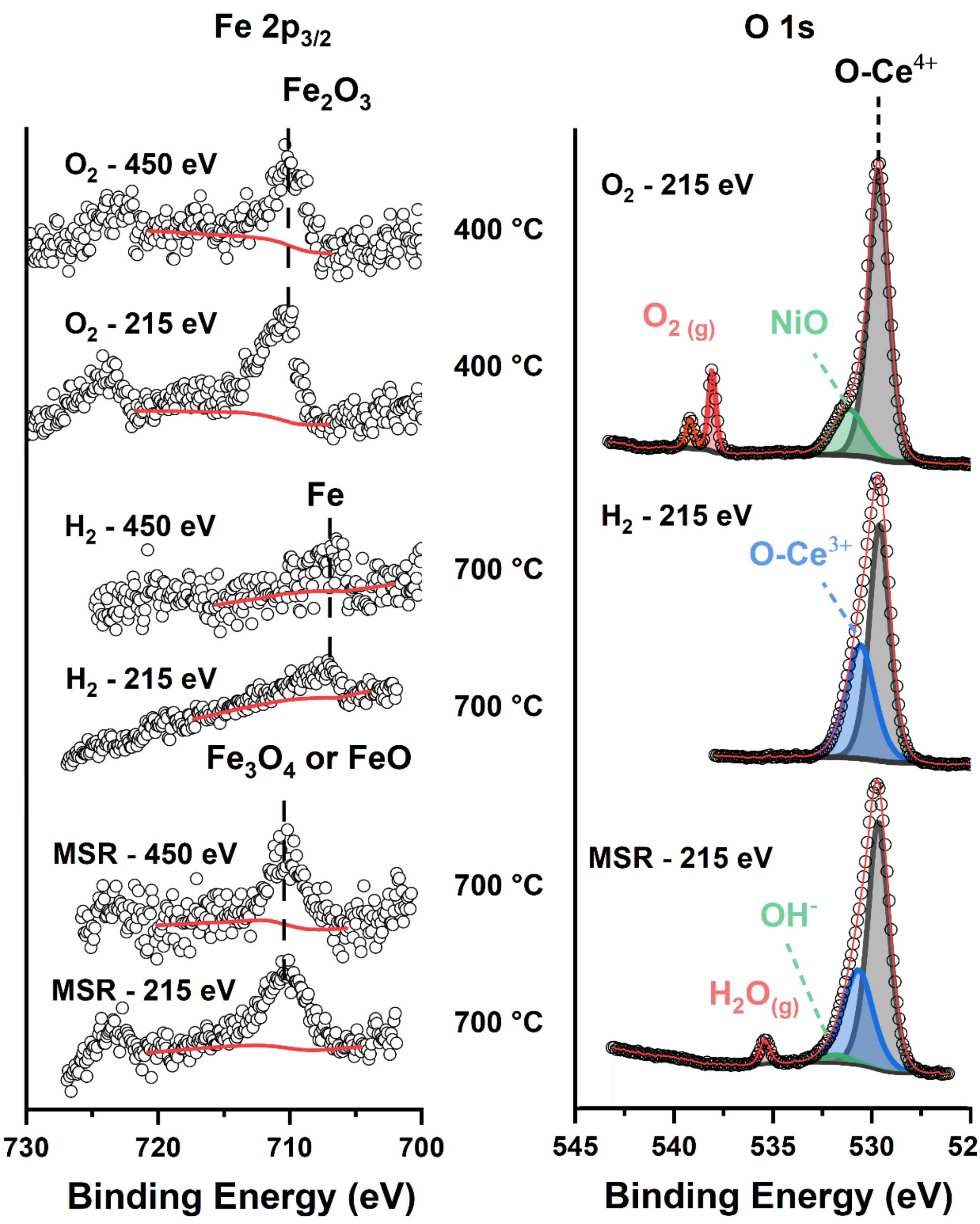


**Figure S11**. NAP-XPS spectra of Fe $2p_{3/2}$ and O 1s of $Fe_{0.1}Ni_{0.9}/CeO_2$-BM under the indicated gas mixtures and temperatures for the two kinetic energies studied at 1 mbar.

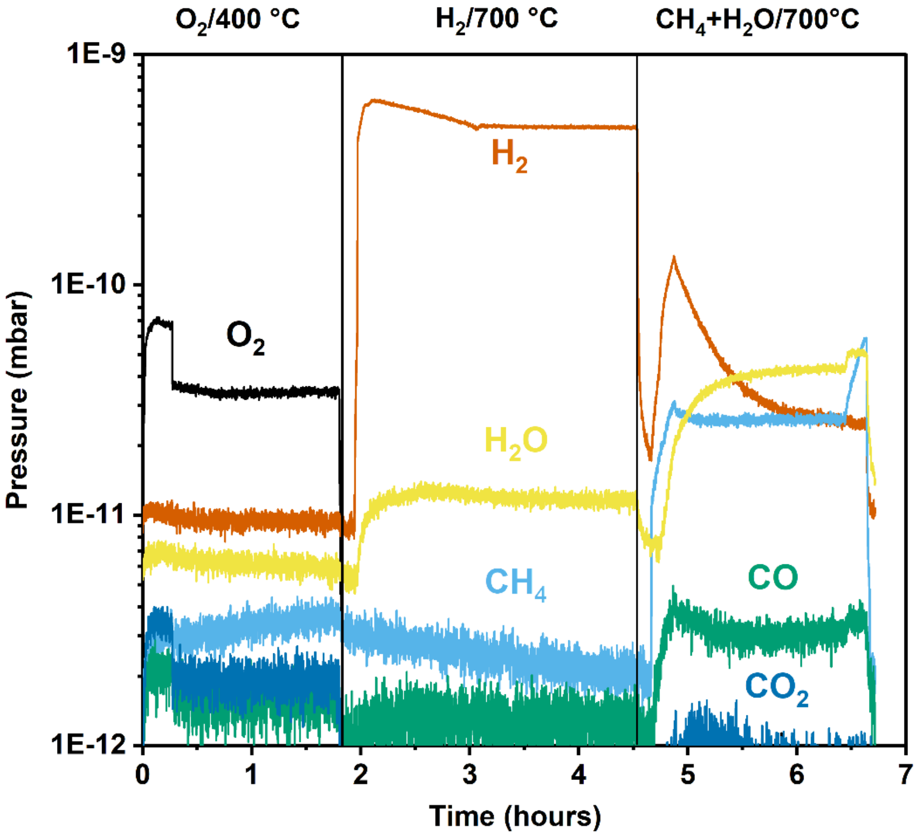


**Figure S12**. Mass spectrometer signals measured during the NAP-XPS measurements of $Fe_{0.1}Ni_{0.9}/CeO_2$-BM under the different reaction mixtures at 1 mbar.

## References


[1] A. J. De Abreu, A. F. Lucrédio, E. M. Assaf, *Fuel Process. Technol.* **2012**, *102*, 140–145.

[2] X. Fang, X. Zhang, Y. Guo, M. Chen, W. Liu, X. Xu, H. Peng, Z. Gao, X. Wang, C. Li, *Int. J. Hydrogen Energy* **2016**, *41*, 11141–11153.

[3] G. H. Lai, J. H. Lak, D. H. Tsai, *ACS Appl. Energy Mater.* **2019**, DOI 10.1021/acsaem.9b01444.

[4] M. Aghayan, D. I. Potemkin, F. Rubio-Marcos, S. I. Uskov, P. V. Snytnikov, I. Hussainova, *ACS Appl. Mater. Interfaces* **2017**, *9*, 43553–43562.

[5] T. Miyata, D. Li, M. Shiraga, T. Shishido, Y. Oumi, T. Sano, K. Takehira, *Appl. Catal. A Gen.* **2006**, *310*, 97–104.

[6] S. C. Baek, K. W. Jun, Y. J. Lee, J. D. Kim, D. Y. Park, K. Y. Lee, in *Res. Chem. Intermed.*, **2012**, pp. 1225–1236.

[7] V. K. Jaiswar, S. Katheria, G. Deo, D. Kunzru, *Int. J. Hydrogen Energy* **2017**, *42*, 18968–18976.
